# N, S-Functionalized Magnetic Rice Husk Biochar for Flucloxacillin Sodium Adsorption: Regeneration and Water-Matrix Effects

**DOI:** 10.3390/ijms27209082

**Published:** 2026-10-13

**Authors:** Prashant Pandey, Manisha Dhiman, Andrzej Białowiec, Amit Pokhriyal, Kanchan Deoli Bahukhandi, Parmjeet Kaur, Chinenye Adaobi Igwegbe

**Affiliations:** 1 Department of Applied Bioeconomy, Wroclaw University of Environmental and Life Sciences, 51-630 Wroclaw, Poland; prashant.pandey@upwr.edu.pl (P.P.); chinenye.igwegbe@upwr.edu.pl (C.A.I.); 2 Institute of Liberal Arts and Humanities (Economics), Chandigarh University, Mohali 140413, Punjab, India; manisha.e19116@cumail.in; 3 Sustainability Cluster, School of Advanced Engineering, UPES University, Dehradun 248001, Uttarakhand, India; amit_enviro2003@yahoo.co.in (A.P.); kanchan@ddn.upes.ac.in (K.D.B.); 4 Uttarakhand Pollution Control Board, Gaura Devi Paryavaran Bhawan, IT Park, Dehradun 248001, Uttarakhand, India; 5 Department of Economics, Lovely Professional University, Phagwara 144401, Punjab, India; parmjeet.28750@lpu.co.in; 6 Department of Chemical Engineering, Nnamdi Azikiwe University, P.M.B. 5025, Awka 420218, Nigeria

**Keywords:** rice husk biochar, magnetic N, S co-doped biochar, flucloxacillin sodium, adsorption, surface functionalization, regeneration, wastewater treatment

## Abstract

Rice husk-derived biochars with progressively modified surface properties were investigated for adsorbing flucloxacillin sodium (FLX-Na) from aqueous solution. Four materials—rice husk biochar (RHB), nitrogen-functionalized RHB (N-RHB), magnetic nitrogen-functionalized RHB (MN-RHB), and magnetic nitrogen/sulfur-functionalized RHB (MN,S-RHB)—were prepared and characterised using morphological, structural, textural, and surface-chemical techniques. The sequential preparation routes produced materials with different surface areas, pore structures, and surface functionalities. FLX-Na adsorption increased in the order RHB < MN-RHB < N-RHB < MN, S-RHB, with the highest equilibrium adsorption capacity obtained for MN,S-RHB (118.6 mg g^−1^). Because chemical activation and a higher carbonisation temperature were introduced only for MN,S-RHB, the higher uptake is interpreted as an effect of the combined modification route rather than being attributed specifically to N/S co-doping. Kinetic and equilibrium data were described using several empirical models, with the pseudo-second-order and Sips models giving the highest coefficients of determination among the tested fits. The adsorption capacity of MN,S-RHB decreased from 84.60 ± 1.30 mg g^−1^ in deionised water to 60.30 ± 1.80 mg g^−1^ in a mixed inorganic/humic-acid matrix, corresponding to 71.3% capacity retention. Spectroscopic changes after adsorption were consistent with changes in the surface chemical environment, while the matrix experiments indicated that coexisting inorganic ions and dissolved organic matter influenced adsorption performance. These results show that the combined physicochemical modification route altered the adsorption behaviour of rice husk-derived biochar under the investigated conditions.

## 1. Introduction

The increasing consumption of pharmaceutical compounds has led to their widespread detection in natural and engineered aquatic environments, raising concerns regarding their persistence and potential ecological impacts [1,2]. Among these contaminants, antibiotics have received considerable attention because of their extensive use in human and veterinary medicine and their continuous release into water bodies through hospital effluents, municipal wastewater, pharmaceutical manufacturing discharges, and agricultural runoff [3]. Conventional wastewater treatment plants are primarily designed to remove biodegradable organic matter and nutrients, and therefore often exhibit limited removal efficiencies for many pharmaceutical compounds. So, residual antibiotics may persist in treated effluents and subsequently enter surface water, groundwater, and soil systems, where they can contribute to the development of antimicrobial resistance and affect aquatic organisms [4]. β-Lactam antibiotics constitute one of the most widely prescribed classes of antimicrobial agents due to their broad-spectrum antibacterial activity and favourable clinical safety profile. This class includes penicillins, cephalosporins, carbapenems, and monobactams, all of which contain a characteristic β-lactam ring responsible for their antibacterial activity [5,6,7]. Although β-lactam antibiotics are generally susceptible to hydrolysis, continuous discharge from anthropogenic sources can maintain measurable concentrations in aquatic environments. Even at relatively low concentrations, prolonged exposure may influence microbial communities and increase the selective pressure for antibiotic-resistant bacteria and resistance genes. These concerns have highlighted the need for effective approaches to reduce the release of antibiotic residues into aquatic systems.

Flucloxacillin sodium (FLX-Na), a semisynthetic isoxazolyl penicillin, is widely prescribed for the treatment of infections caused by penicillinase-producing *Staphylococcus* species and other Gram-positive bacteria [8,9]. Owing to its extensive clinical use, incomplete metabolism in the human body, and subsequent excretion, FLX-Na may enter municipal wastewater and healthcare effluents. The molecular structure of FLX-Na contains aromatic rings together with amino, carboxyl, amide, and β-lactam functional groups, enabling multiple interactions with solid surfaces through hydrogen bonding, electrostatic attraction, and electron donor-acceptor interactions. These structural characteristics also influence its environmental behaviour and present challenges for complete removal using conventional treatment processes.

Several treatment technologies, including advanced oxidation processes, membrane filtration, biological degradation, photocatalysis, electrochemical oxidation, coagulation-flocculation, and adsorption, have been investigated for the removal of pharmaceutical contaminants from water [10,11,12,13]. Advanced oxidation and photocatalytic processes can effectively degrade a range of pharmaceuticals; however, their application is often associated with relatively high energy requirements, catalyst recovery challenges, and the possible formation of transformation products. Membrane-based technologies generally provide high separation efficiencies but are limited by membrane fouling, concentrate management, and operational costs. Biological treatment processes may partially remove certain antibiotics, although removal efficiency depends on the physicochemical properties of the target compound and the activity of microbial communities [14,15,16]. Thus, adsorption continues to receive considerable attention because of its operational simplicity, adaptability to different water matrices, and the availability of diverse adsorbent materials. The adsorption performance of a solid material is strongly influenced by its specific surface area, pore structure, surface chemistry, and the accessibility of active adsorption sites [17,18]. Activated carbons remain among the most effective adsorbents for pharmaceutical removal; however, their relatively high production cost has encouraged the development of alternative adsorbents derived from renewable biomass resources [19]. Agricultural residues represent an abundant and inexpensive source of carbonaceous materials and provide opportunities for converting biomass waste into value-added functional adsorbents within the framework of circular resource utilisation [20,21,22]. Among these materials, rice husk has attracted increasing interest because of its global availability, high carbon content, naturally occurring silica, and suitability for thermochemical conversion into porous carbon materials.

However, adsorption experiments conducted in simplified aqueous systems do not necessarily represent the conditions encountered in natural waters and wastewater, where dissolved inorganic ions and organic matter may coexist with pharmaceutical contaminants. The composition of the aqueous phase can influence adsorption through changes in ionic strength, competition for available adsorption sites, and interactions between dissolved species, the adsorbent surface, and the target compound. Monovalent and divalent ions may therefore affect adsorption differently, while the combined presence of several ions may produce a different response from that observed with individual electrolytes. Dissolved organic matter, such as humic substances, may also influence adsorption by interacting with the adsorbent surface and/or the dissolved antibiotic. Accordingly, evaluation under controlled water-matrix conditions can provide additional information on how adsorption performance changes with increasing aqueous-phase complexity. Such experiments do not substitute for studies using authentic environmental water or wastewater, but provide a controlled approach for examining the influence of selected inorganic and organic constituents on FLX-Na adsorption.

Pyrolysis of rice husk produces biochar containing carbonaceous domains together with residual silica inherited from the original biomass. Although pristine biochar possesses adsorption capability, its relatively limited surface area and restricted surface functionality often constrain its adsorption performance toward complex organic contaminants [23]. Consequently, several modification strategies have been explored to improve the physicochemical properties of biomass-derived biochars. Chemical activation using alkaline or acidic activating agents can promote pore development and increase accessible surface area, whereas heteroatom doping introduces additional surface functionalities capable of interacting with polar organic molecules [24]. In particular, nitrogen-containing functional groups have been reported to modify the electronic structure of carbon materials and provide additional adsorption sites through hydrogen bonding and acid-base interactions [25]. Sulfur incorporation can further alter surface polarity and contribute additional chemically active sites, while magnetic functionalisation using iron oxides enables rapid separation of the adsorbent from aqueous media after treatment [26,27]. Recent studies have demonstrated that combining chemical activation with heteroatom doping can improve the adsorption behaviour of biomass-derived carbons toward pharmaceutical compounds. Nevertheless, most reported investigations have focused on single-modification strategies or individual adsorbent classes. Comparative studies examining the progressive influence of sequential modification from pristine biochar to heteroatom-doped, magnetically functionalised, and dual-doped magnetic biochars remain relatively limited. Furthermore, understanding how these modifications influence the structural, textural, and surface chemical properties of rice husk-derived adsorbents and how these changes relate to adsorption behaviour remains an active area of investigation.

In the present study, rice husk was utilised as a renewable carbon precursor to prepare four adsorbents with progressively different physicochemical characteristics, namely raw rice husk biochar (RHB), nitrogen-doped rice husk biochar (N-RHB), magnetic nitrogen-doped rice husk biochar (MN-RHB), and magnetic nitrogen and sulfur co-doped rice husk biochar (MN,S-RHB). This study fits within an established body of work on heteroatom-doped and magnetic biochars for antibiotic removal. Recent reports have examined N,S-co-doped magnetic biochars for antibiotic adsorption and N, S-functionalised magnetic biochars for antibiotic transformation, while recent reviews have summarised N-doped magnetic biochars for antibiotic mitigation. The present work therefore focuses on FLX-Na adsorption, the progressive comparison of four rice-husk-derived materials, and the effect of controlled inorganic and dissolved-organic matrix constituents. The adsorbents were synthesised through carbonisation followed by heteroatom incorporation, chemical activation, and magnetic functionalisation, but differences in activation and carbonisation conditions between materials limit the ability to assign effects to individual modifications.

The adsorption performance of the prepared materials toward FLX-Na (details of the antibiotics are summarised in Table 1) was evaluated through batch adsorption experiments. Adsorption kinetics, equilibrium isotherms, and thermodynamic parameters were analysed to investigate the adsorption behaviour of the prepared materials. In addition, the adsorption mechanism was interpreted by integrating the experimental adsorption data with the structural and surface chemical information obtained from the characterisation analyses. The findings of this study provide insight into how sequential physicochemical modification influences the adsorption characteristics of rice husk-derived biochars and may support the development of biomass-based adsorbents for the removal of antibiotic contaminants from aqueous systems.

## 2. Results

### 2.1. Characterizations

#### 2.1.1. Morphological and Elemental Characterisation of Rice Husk-Derived Adsorbents

**Figure 1:**
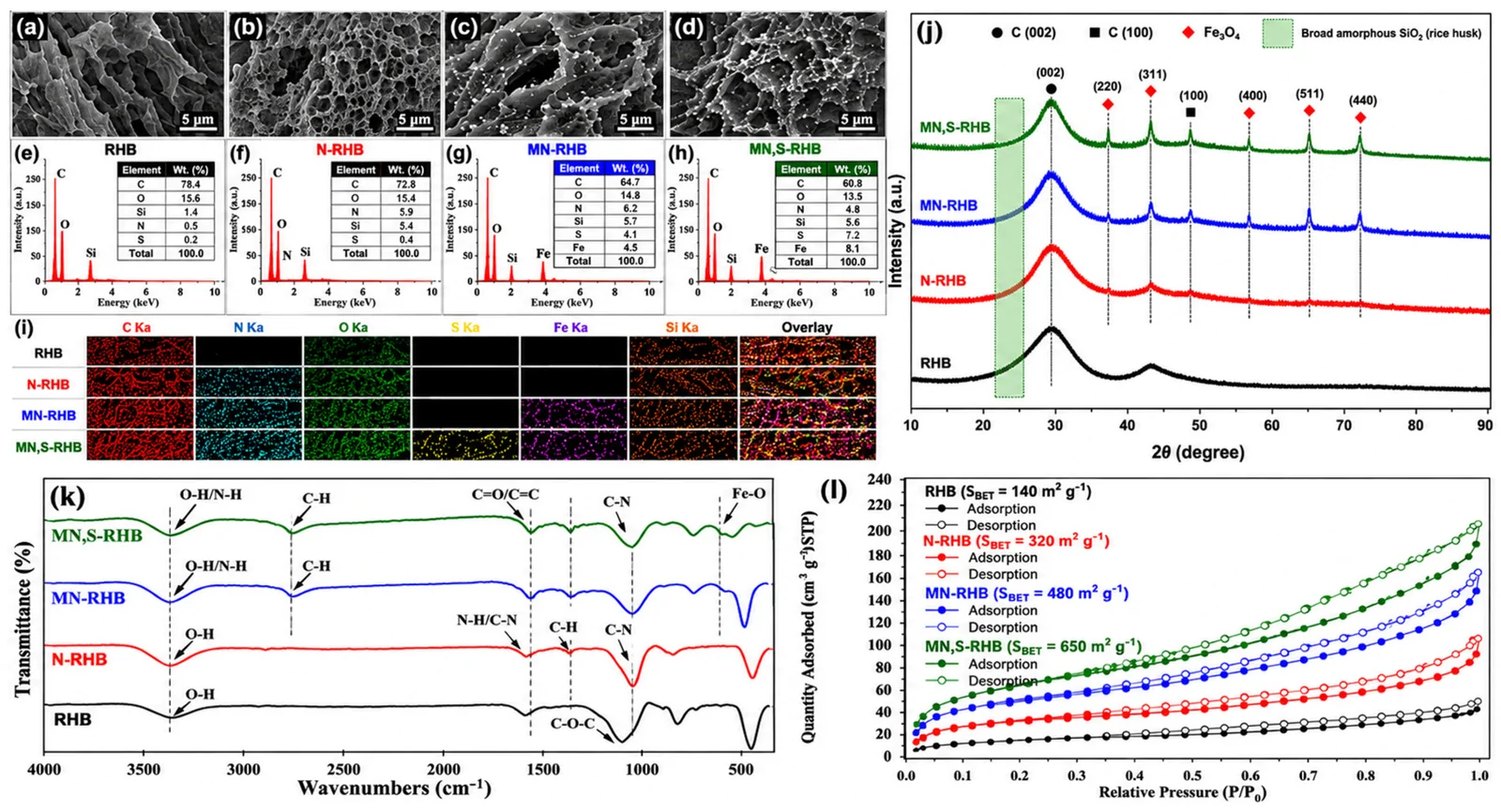
Characterisation of RHB, N-RHB, MN-RHB, and MN,S-RHB: (**a**–**d**) SEM images showing surface morphology; (**e**–**h**) EDS spectra and corresponding elemental compositions; (**i**) elemental mapping showing the distribution of C, N, O, S, Fe, and Si; (**j**) XRD patterns identifying the carbonaceous and Fe_3_O_4_ phases; (**k**) FTIR spectra showing the principal surface functional groups; and (**l**) N_2_ adsorption–desorption isotherms at 77 K showing Type IV(a) behaviour with H4-type hysteresis.

The morphology, elemental composition, crystalline structure, surface functional groups, and textural properties of RHB, N-RHB, MN-RHB, and MN, S-RHB were investigated using SEM-EDS, XRD, FTIR, and N_2_ adsorption-desorption analysis (Figure 1; Supplementary Table S2). SEM images (Figure 1a–d) presented a relatively compact and irregular surface for RHB, whereas N-RHB exhibited a more developed porous surface with interconnected cavities. MN-RHB retained the porous carbon framework and showed dispersed approximately spherical particles on the surface, consistent with the incorporation of the iron-containing phase. MN,S-RHB showed interconnected surface cavities with dispersed iron-containing particles. The observed morphological differences were consistent with the measured textural properties. The EDS spectra (Figure 1e–h) display carbon and oxygen as the major detected elements, together with silicon associated with the rice-husk-derived material. Nitrogen was detected in N-RHB, MN-RHB, and MN,S-RHB, while iron was detected in MN-RHB and MN,S-RHB. Sulfur was detected in MN,S-RHB, consistent with the use of thiourea as the sulfur-containing precursor. Elemental mapping showed carbon and oxygen throughout the samples, with nitrogen, iron, and sulfur detected in the corresponding modified materials.

The XRD patterns (Figure 1j) present broad reflections at approximately 24–25° and 43–44°, assigned to the (002) and (100) planes of turbostratic carbon, respectively. RHB also present a broad feature at 20–23°, attributed to amorphous silica originating from the rice husk precursor. MN-RHB and MN,S-RHB exhibited additional reflections at approximately 30.1°, 35.5°, 43.2°, 53.5°, 57.0°, and 62.6°, corresponding to the (220), (311), (400), (422), (511), and (440) planes of magnetite (Fe_3_O_4_; JCPDS No. 19-0629), respectively. The broad carbon reflections remained visible after magnetic modification. For MN,S-RHB, d_002_ increased from 3.56 to 3.64 Å, while L_c_ increased from 1.92 to 2.87 nm and the crystallinity index from 28.6% to 44.6%. No additional crystalline sulfur-containing phase was detected.

The FTIR spectra of RHB, N-RHB, MN-RHB, and MN,S-RHB were recorded over the 4000–500 cm^−1^ range, with the wavenumber decreasing from left to right. A broad band at approximately 3380 cm^−1^ was observed for all samples and was assigned to O-H/N-H stretching vibrations. A feature near 2765 cm^−1^ was associated with C-H stretching. The spectra also exhibited bands at 1550 and 1615 cm^−1^ region, which are assigned to C=O/C=C-related vibrations and, for the nitrogen-containing samples, N-H/C=N-related contributions. The band near 1390 cm^−1^ was attributed to C-H bending/deformation. A prominent feature around 1050 cm^−1^ was assigned to overlapping C-O/C-O-C and C-N vibrations. For MN-RHB and MN,S-RHB, an additional band in the low-wavenumber region at approximately 610 cm^−1^ was observed and attributed to Fe-O vibrations. Hence, the spectra show changes in the surface functional-group environment following nitrogen incorporation and magnetic modification. The observed spectral positions of these bands are consistent with reported studies [28,29,30]. The FTIR peak positions, corresponding vibrational assignments, and associated elements of RHB, N-RHB, MN-RHB, and MN, S-RHB are summarised in SI- Table S2.

The N_2_ adsorption–desorption isotherms measured at 77 K (Figure 1l) demonstrate type IV(a)-type behaviour with hysteresis, indicating the presence of mesoporous characteristics in the investigated materials. The BET surface area increased from 140 m^2^ g^−1^ for RHB to 320, 480, and 650 m^2^ g^−1^ for N-RHB, MN-RHB, and MN,S-RHB, respectively. The corresponding total pore volumes were 0.082, 0.165, 0.248, and 0.325 cm^3^ g^−1^, respectively. These results indicate an increase in accessible surface area and pore volume after successive modification steps. The pore-size analysis showed pores predominantly within the mesoporous range. The morphological changes observed by SEM accompanied the changes in textural properties. Hence, the obtained results demonstrate differences in the textural characteristics of RHB and the modified rice-husk-derived biochars.

#### 2.1.2. Additional Characterisation of the Adsorbents

**Figure 2:**
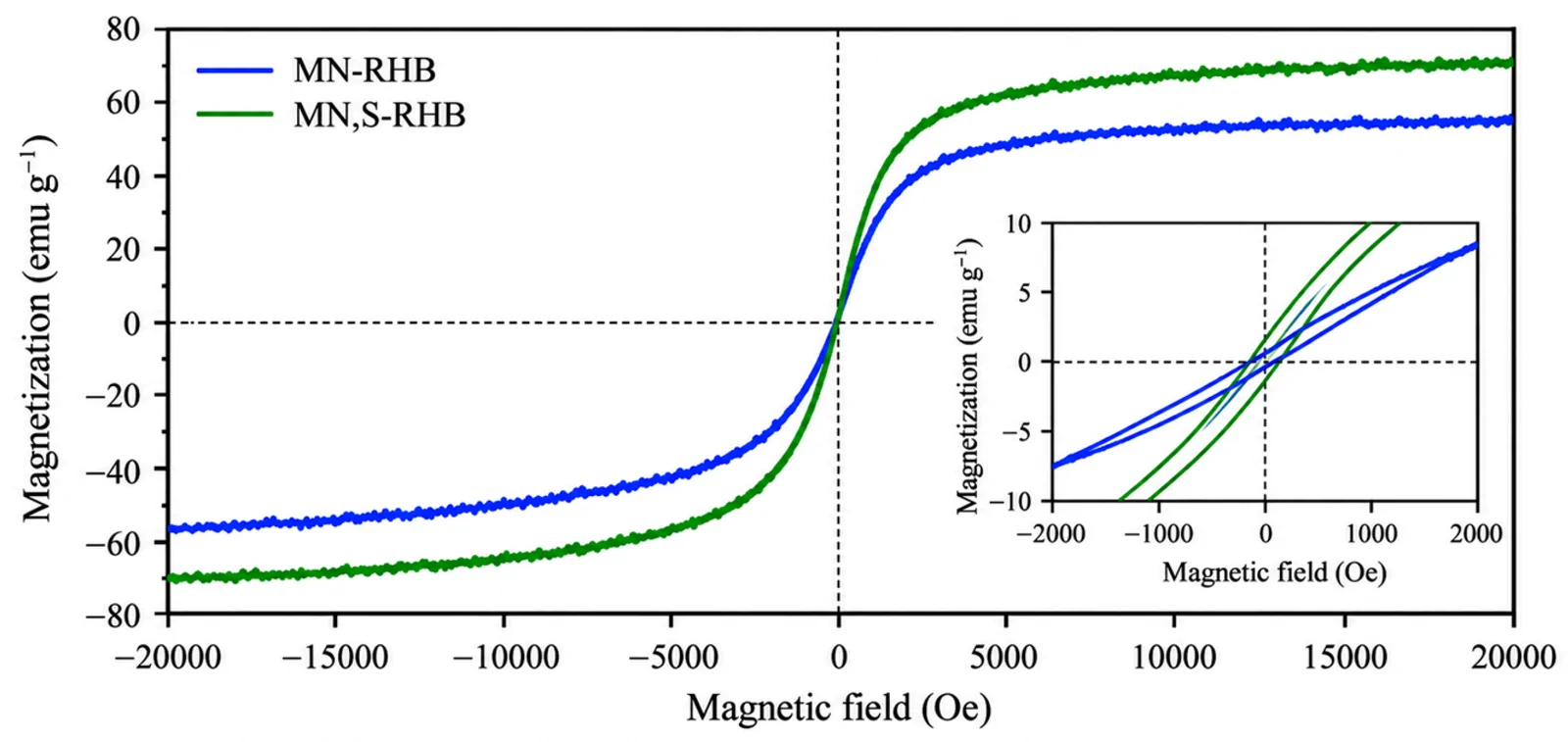
VSM hysteresis curves of MN-RHB and MN,S-RHB. The inset shows the low-field region used to evaluate remanent magnetization and coercivity.

Additional characterisation was performed to assess the carbon structural environment, bulk elemental composition, surface-charge behaviour, magnetic properties, and Fe loading of the prepared materials (presented in Figure 2).

The magnetic response of MN-RHB and MN,S-RHB was evaluated by vibrating sample magnetometry (VSM). Both materials exhibited magnetic hysteresis behaviour. MN-RHB showed a saturation magnetization (M_s_) of 55.2 ± 1.8 emu g^−1^, a remanent magnetization (M_r_) of 2.6 ± 0.3 emu g^−1^, and a coercivity (Hc) of 84 ± 12 Oe. For MN,S-RHB, the corresponding values were 68.7 ± 2.1 emu g^−1^, 3.8 ± 0.4 emu g^−1^, and 110 ± 15 Oe, respectively (Table 2). The VSM results confirm measurable magnetic response for both magnetically modified adsorbents.

**Figure 3:**
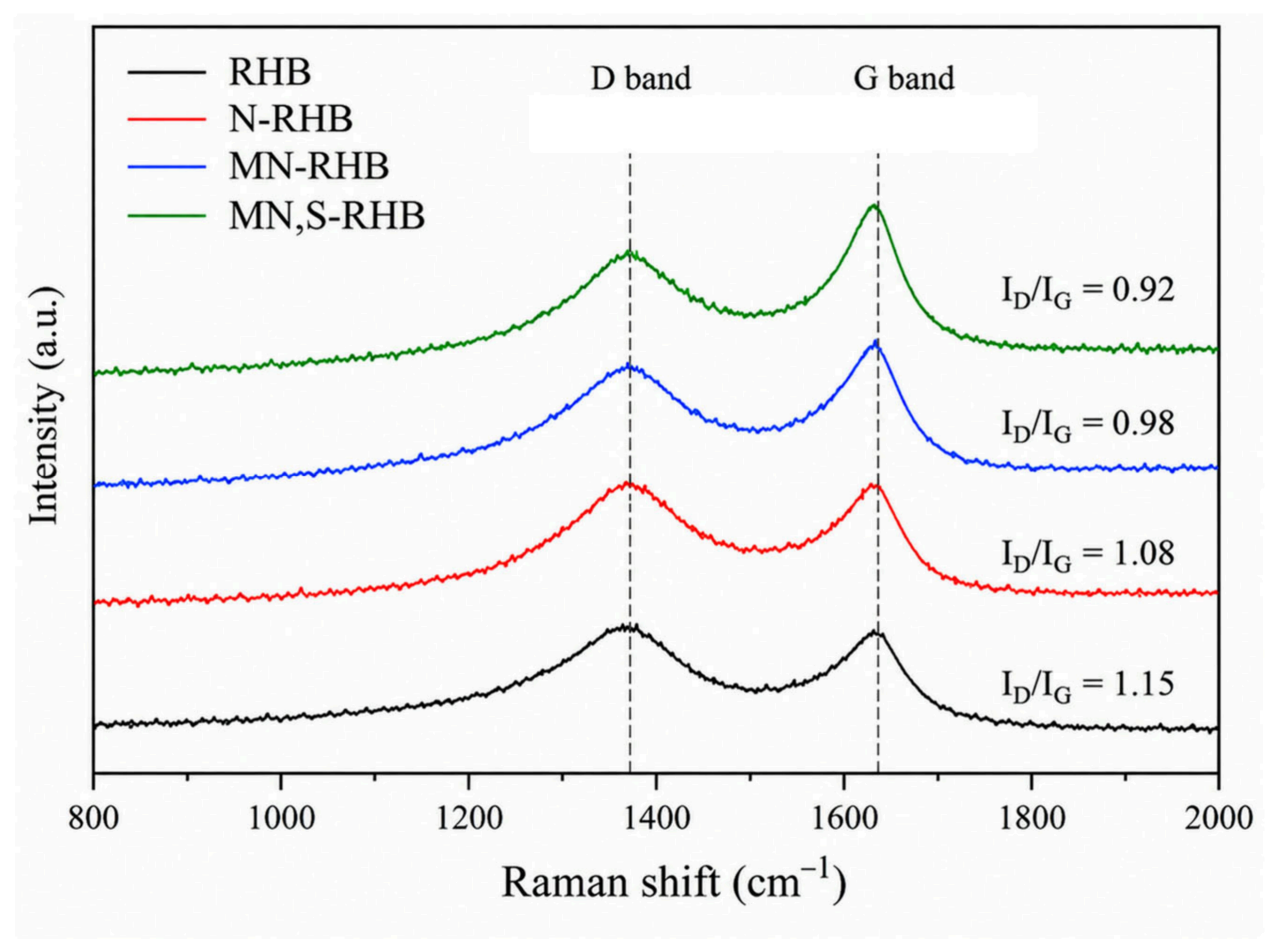
Raman spectra of RHB, N-RHB, MN-RHB, and MN,S-RHB. The characteristic D and G bands are observed at approximately 1350 and 1640 cm^−1^, respectively. Spectra are vertically offset for clarity. The corresponding IID/IG ratios are indicated for each material.

The Raman spectra of RHB, N-RHB, MN-RHB, and MN,S-RHB exhibited characteristic D and G bands at approximately 1350 and 1640 cm^−1^, respectively. The calculated ID/IG ratios were 1.15, 1.08, 0.98, and 0.92 for RHB, N-RHB, MN-RHB, and MN,S-RHB, respectively (presented in Figure 3). The variation in ID/IG indicates changes in the relative contribution of disordered and graphitic carbon domains following the sequential modification steps. The Raman results therefore indicate changes in the carbon structural environment across the material series. However, the observed differences cannot be attributed exclusively to N/S incorporation because the preparation routes also involved changes in magnetic functionalisation, activation, and carbonisation conditions.

**Table 2:** Magnetic parameters of MN-RHB and MN,S-RHB obtained from VSM analysis. Values are mean ± standard deviation (*n* = 3). M_s_, saturation magnetization; M_r_, remanent magnetization; Hc, coercivity.

Material	M_s_ (emu g^−1^)	M_r_ (emu g^−1^)	Hc (Oe)
**MN-RHB**	55.2 ± 1.8	2.6 ± 0.3	84 ± 12
**MN,S-RHB**	68.7 ± 2.1	3.8 ± 0.4	110 ± 15

**Figure 4:**
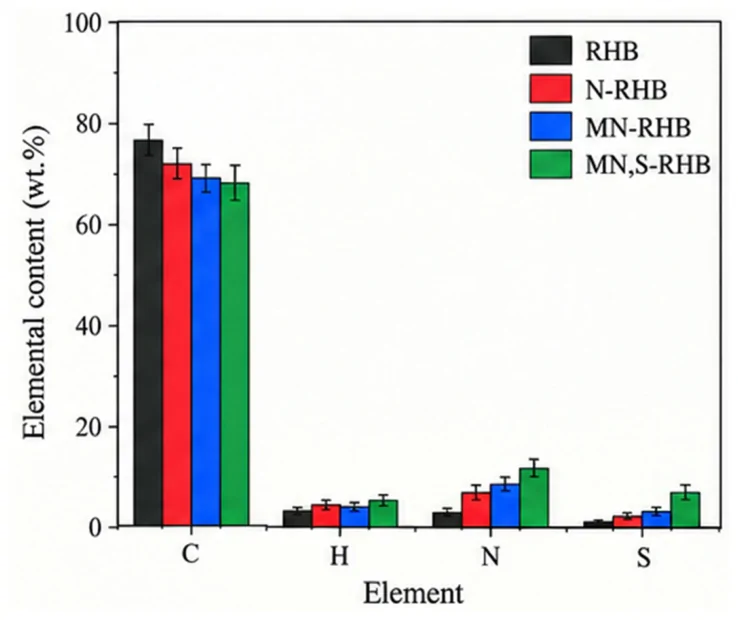
CHNS elemental composition of RHB, N-RHB, MN-RHB, and MN,S-RHB. Error bars represent the standard deviation of replicate measurements, where applicable.

CHNS elemental analysis was performed to examine the bulk elemental composition of RHB, N-RHB, MN-RHB, and MN,S-RHB, presented in Figure 4. Carbon was the predominant element in all four materials, while the relative contents of hydrogen and nitrogen varied among the sequentially modified adsorbents. Nitrogen was detected in N-RHB, MN-RHB, and MN,S-RHB, consistent with the use of nitrogen-containing precursors during the modification process. Sulfur was detected in MN,S-RHB, whereas its contribution was negligible in the other materials, consistent with the use of thiourea in its preparation. The CHNS results therefore indicate changes in the bulk elemental composition following the sequential modification of the rice-husk-derived carbon. The observed elemental trends were qualitatively consistent with the EDS results, which were used as a complementary localized compositional measurement.

**Figure 5:**
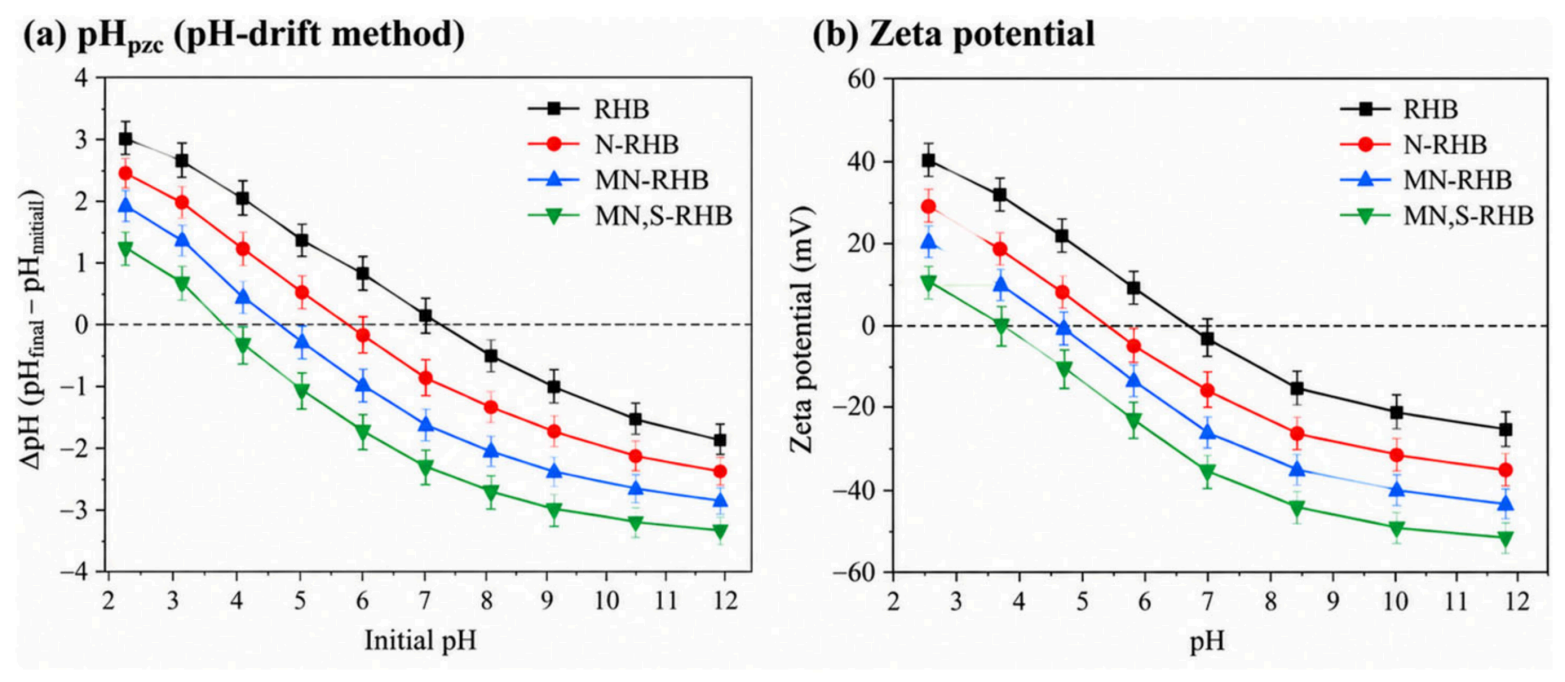
(**a**) pH_PZC_ determined by the pH-drift method and (**b**) zeta potential as a function of pH for RHB, N-RHB, MN-RHB and MN,S-RHB. The pH_PZC_ values were determined from the intersection of ΔpH with zero, while the isoelectric points were estimated from the pH at which the zeta potential crossed zero.

The surface-charge characteristics of the four adsorbents were evaluated using pH-drift and zeta-potential measurements (shown in Figure 5). The pH_PZC_ values obtained by the pH-drift method were 7.4 ± 0.1, 5.6 ± 0.1, 4.9 ± 0.1 and 4.3 ± 0.1 for RHB, N-RHB, MN-RHB and MN,S-RHB, respectively. The corresponding isoelectric points estimated from the zero-crossing of the zeta-potential curves were 7.3 ± 0.1, 5.5 ± 0.1, 4.7 ± 0.1, and 4.2 ± 0.1, respectively. The close correspondence between the two measurements indicates that the surface-charge characteristics changed progressively across the material series. These measurements were used to describe surface-charge behaviour and were not treated as direct proof of a specific adsorption mechanism.

**Figure 6:**
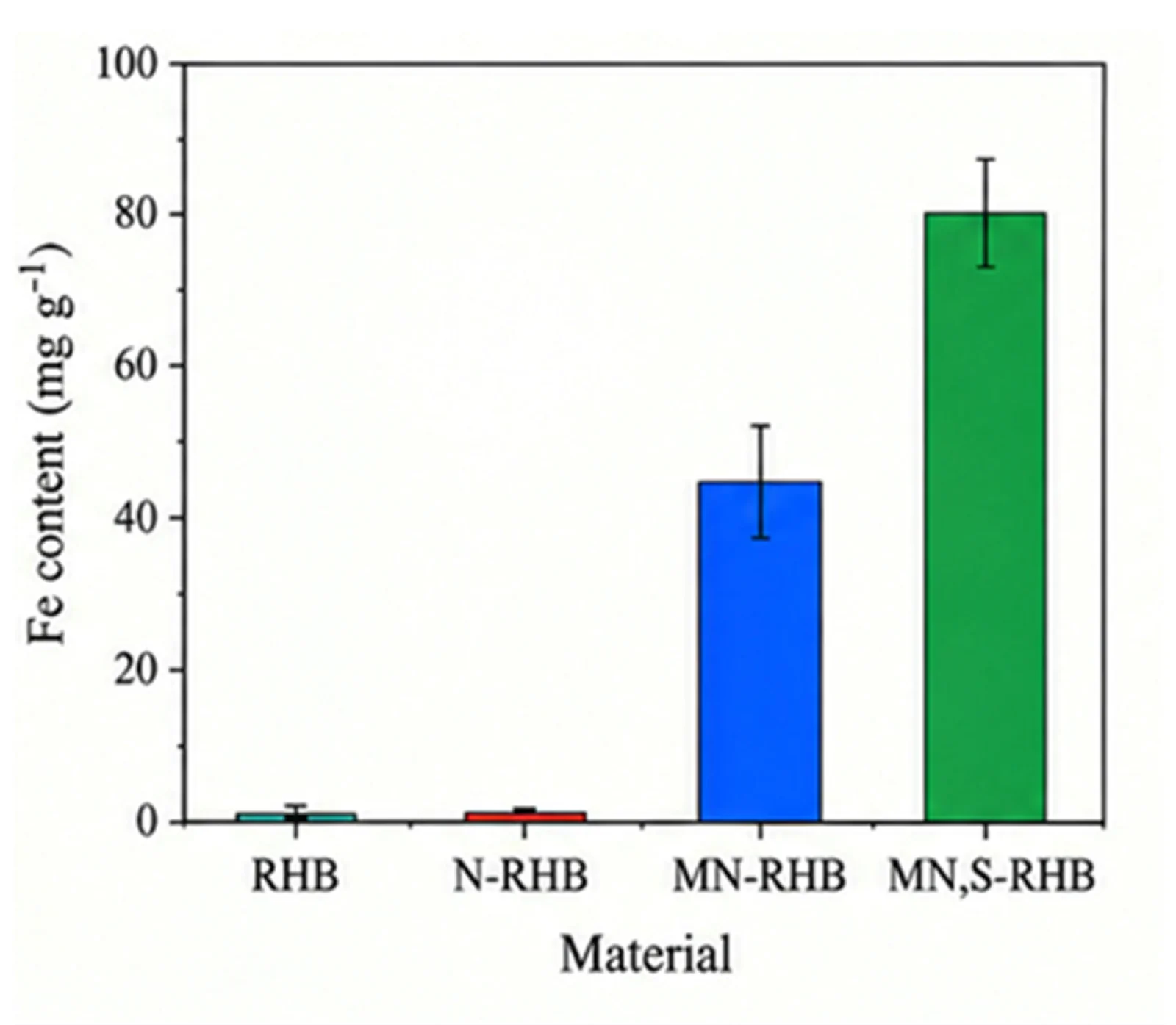
Fe content of RHB, N-RHB, MN-RHB, and MN,S-RHB determined by ICP-OES. Error bars represent the standard deviation of replicate measurements, where applicable.

ICP-OES quantified the Fe content of the prepared adsorbents to assess the extent of Fe incorporation following magnetic modification. RHB and N-RHB contained only low levels of Fe, whereas MN-RHB and MN,S-RHB contained substantially higher Fe contents. The higher Fe contents of the latter two materials are consistent with the introduction of the Fe-containing magnetic component during their preparation, presented in Figure 6. The ICP-OES results provide quantitative bulk information on Fe content and complement the Fe signals observed by EDS. Because ICP-OES measures total Fe, the values were considered together with XRD and FTIR characterisation when evaluating the magnetic phase.

### 2.2. Adsorption Studies

#### 2.2.1. Effect of Contact Time and Kinetics

The effect of contact time on FLX-Na adsorption by RHB, N-RHB, MN-RHB and MN,S-RHB was evaluated under the experimental conditions described in the methodology. Briefly, 250 mL of FLX-Na solution at an initial concentration of 200 mg L^−1^ was mixed with 0.5 g of adsorbent and stirred at 200 rpm at 298 K. Aliquots were collected at predetermined time intervals to monitor changes in adsorption capacity over time.

**Figure 7:**
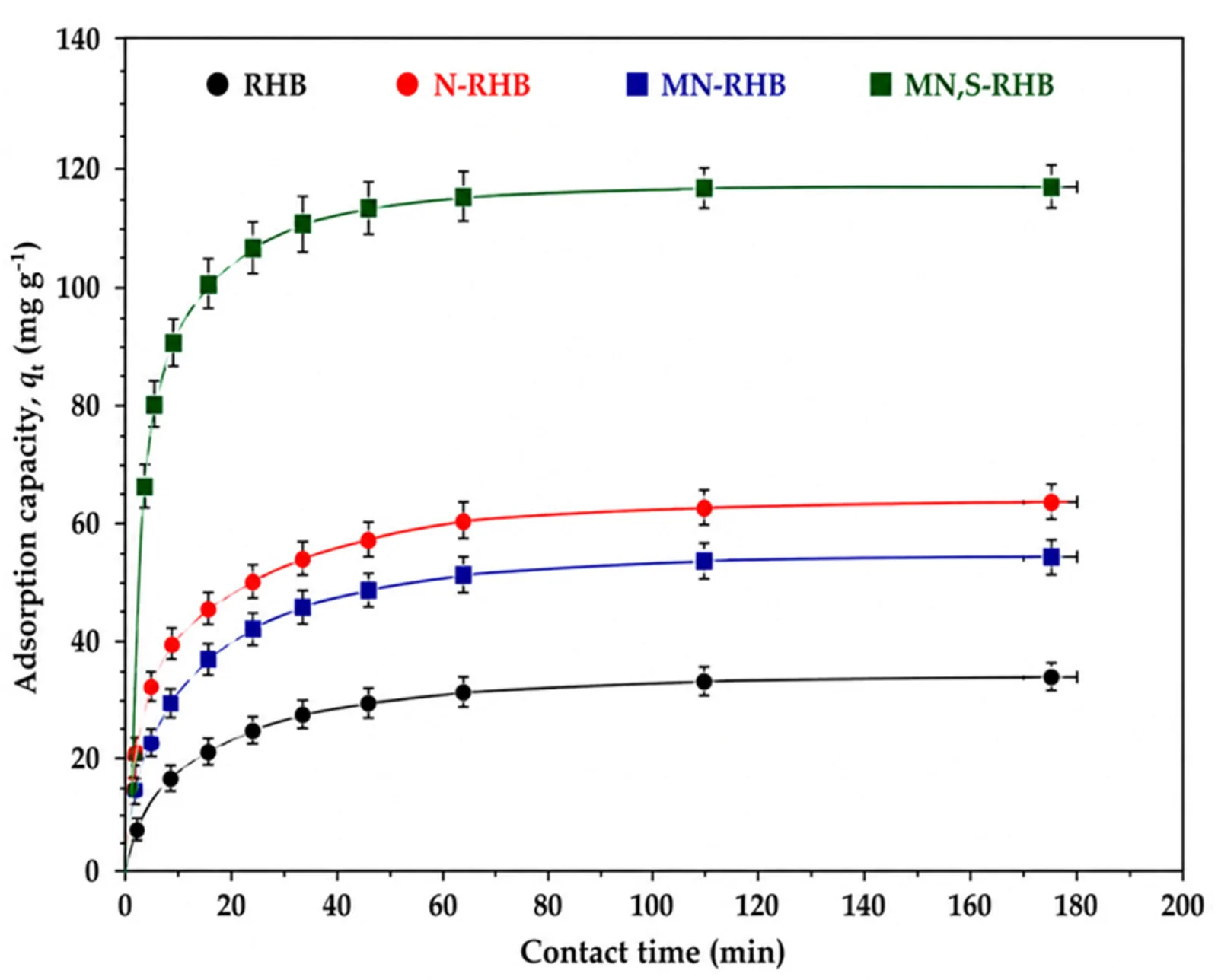
Effect of contact time on the adsorption of FLX-Na onto RHB, N-RHB, MN-RHB, and MN,S-RHB.

All 04 adsorbents displayed an increase in adsorption capacity with increasing contact time, followed by a progressively smaller change in q_t_ as the adsorption process approached a plateau (Figure 7). The initial increase was associated with the availability of accessible adsorption sites and the concentration gradient between FLX-Na in solution and the adsorbent surface. With increasing contact time, occupation of available sites and reduction in the concentration gradient resulted in a lower rate of uptake. The adsorption rate differed among the materials. RHB showed the lowest uptake throughout the investigated period, reaching an equilibrium adsorption capacity of 34.8 mg g^−1^ at approximately 180 min. N-RHB exhibited a faster initial uptake and reached 62.4 mg g^−1^ at approximately 120 min. MN-RHB reached 54.8 mg g^−1^ at approximately 120 min. MN,S-RHB showed the highest uptake, increasing from 66.5 mg g^−1^ at 5 min to 80.3, 91.2, 100.5, 106.8, 111.2 and 114.3 mg g^−1^ at 10, 20, 30, 45, 60 and 90 min, respectively, before approaching a plateau at approximately 118.6 mg g^−1^. For RHB, N-RHB and MN-RHB, the relatively slower approach to the plateau compared with MN,S-RHB indicates differences in the rate at which FLX-Na accessed available adsorption sites. The faster uptake observed for MN,S-RHB is consistent with its higher BET surface area (650 m^2^ g^−1^) and pore volume (0.325 cm^3^ g^−1^) compared with RHB (140 m^2^ g^−1^ and 0.082 cm^3^ g^−1^), N-RHB (320 m^2^ g^−1^ and 0.165 cm^3^ g^−1^) and MN-RHB (480 m^2^ g^−1^ and 0.248 cm^3^ g^−1^). The surface and structural characterisation provides additional context for these differences. SEM showed changes in the surface morphology following nitrogen modification and magnetic functionalisation, while FTIR and EDS indicated changes in surface composition. For MN-RHB and MN,S-RHB, the FTIR spectra also showed Fe-O vibrations associated with Fe_3_O_4_. These changes, together with the increase in pore development, can influence the accessibility of FLX-Na to the adsorbent surface.

The contact-time data were fitted using the pseudo-first-order (PFO), pseudo-second-order (PSO), and intraparticle-diffusion models. The kinetic equations and parameter definitions follow the methodology Section. The models were compared using the calculated equilibrium adsorption capacity and coefficient of determination (R^2^), details of the determined values are summarised in Table 3.

The PFO model gave calculated q_e_ values of 28.5, 53.1, 47.6 and 103.2 mg g^−1^ for RHB, N-RHB, MN-RHB and MN,S-RHB, respectively, with corresponding R^2^ values of 0.921, 0.944, 0.937 and 0.958. The PSO model produced calculated q_e_ values of 33.4, 60.3, 53.1 and 114.4 mg g^−1^, with R^2^ values of 0.991, 0.995, 0.993 and 0.998, respectively. Thus, the PSO model provided higher R^2^ values and calculated capacities closer to the experimental values than the PFO model for all four adsorbents. The corresponding PFO rate constants were 0.015, 0.022, 0.018 and 0.031 min^−1^, whereas the PSO rate constants were 0.0028, 0.0045, 0.0039 and 0.0084 g mg^−1^ min^−1^ for RHB, N-RHB, MN-RHB and MN,S-RHB, respectively. The higher PSO R^2^ values indicate that this model provided a better empirical description of the experimental kinetic profiles under the investigated conditions. This result should be interpreted as a comparison of model fit and does not, by itself, establish a chemisorption mechanism.

The Elovich model gave R^2^ values of 0.945, 0.963, 0.958 and 0.982 for RHB, N-RHB, MN-RHB and MN,S-RHB, respectively. The corresponding α values were 4.18, 9.64, 8.23 and 21.85 mg g^−1^ min^−1^, while the β values were 0.153, 0.097, 0.104 and 0.061 g mg^−1^, respectively. The relatively good fits indicate that the Elovich equation also described the time-dependent adsorption behaviour, particularly for MN,S-RHB. However, these parameters primarily describe the empirical adsorption kinetics and should not be used alone to assign a specific molecular mechanism.

The Weber-Morris intraparticle-diffusion model yielded k_id_ values of 2.48, 3.92, 3.61 and 6.88 mg g^−1^ min^−1/2^ for RHB, N-RHB, MN-RHB and MN,S-RHB, respectively. The corresponding intercepts (CC) were 6.4, 11.2, 9.6 and 18.4 mg g^−1^, with R^2^ values of 0.931, 0.946, 0.939 and 0.968, respectively. The non-zero intercepts indicate that the fitted intraparticle-diffusion lines do not pass through the origin. Therefore, intraparticle diffusion contributed to FLX-Na uptake but was not the only transport contribution represented by the model.

The kinetic results can be considered together with the equilibrium and characterisation results. MN,S-RHB showed the highest adsorption capacity and the highest k_id_ value, together with the largest BET surface area and pore volume. N-RHB presented better equilibrium uptake than MN-RHB despite its lower BET surface area, indicating that surface modification and pore structure both contribute to the observed adsorption behaviour. This interpretation is also consistent with the characterisation results showing changes in surface functional groups and the presence of Fe_3_O_4_ in the magnetic materials. Among the investigated materials, MN,S-RHB showed the highest adsorption capacity and fastest approach to its plateau. The kinetic modelling showed that PSO provided the highest R^2^ values for all four adsorbents, while the intraparticle-diffusion analysis indicated that pore diffusion contributed to, but did not solely control, the adsorption process.

To further distinguish the relative contributions of external film diffusion and intraparticle diffusion, the Boyd diffusion model was applied. The Boyd plots did not pass through the origin for any of the prepared adsorbents, suggesting that external film diffusion contributed to the overall adsorption rate under the investigated conditions. This observation is consistent with the Weber-Morris analysis, where the intraparticle diffusion plots also deviated from the origin, indicating that intraparticle diffusion was not the sole rate-controlling step. The relatively higher specific surface area and hierarchical pore structure of MN,S-RHB, as revealed by BET and SEM analyses, are expected to facilitate the diffusion of FLX-Na molecules into the internal pore network after transport across the external boundary layer. Therefore, the adsorption process appears to involve both external mass transfer and intraparticle diffusion, with their relative contributions varying throughout the adsorption process.

#### 2.2.2. Effect of Solution Ph

The effect of solution pH on FLX-Na adsorption was investigated over the pH range of 2–12 at an initial FLX-Na concentration of 200 mg L^−1^, following the same adsorbent dosage and contact time of 120 min, and at 298 K. The graph for the effect of pH can be accessed from Figure S2 in the Supplementary Information. The adsorption capacity varied with pH for all four adsorbents, indicating that solution pH influenced FLX-Na uptake. This behaviour can be associated with changes in FLX-Na speciation and the protonation state and surface charge of the adsorbents. The observed pH dependence is consistent with the different surface chemistries of RHB, N-RHB, MN-RHB, and MN,S-RHB resulting from nitrogen incorporation, magnetic functionalisation, and N/S co-doping. The pH-dependent adsorption behaviour can also be considered in relation to the surface characteristics obtained from the characterisation analyses. RHB exhibited the lowest surface area and pore volume, whereas MN,S-RHB possessed the highest BET surface area (650 m^2^ g^−1^) and pore volume (0.325 cm^3^ g^−1^). FTIR and EDS analyses indicated the presence of oxygen- and nitrogen-containing functionalities in the modified biochars, with additional sulfur and iron detected in MN,S-RHB. These differences in surface chemistry and textural properties can influence the interaction of FLX-Na with the adsorbent surface under different pH conditions.

#### 2.2.3. Effect of Initial Flx-Na Concentration

The effect of initial FLX-Na concentration was investigated to determine its influence on the equilibrium adsorption capacity of the prepared biochars. Increasing the initial FLX-Na concentration increased the amount of FLX-Na available for transfer from the aqueous phase to the adsorbent surface. The adsorption capacity therefore increased with increasing concentration, while the rate of increase became less pronounced at higher concentrations, consistent with progressive occupation of available adsorption sites. The graph of the study is presented in Supplementary Information as Figure S3. The adsorption response differed among the four adsorbents and was consistent with their physicochemical characteristics. RHB, which had a BET surface area of 140 m^2^ g^−1^ and pore volume of 0.082 cm^3^ g^−1^, showed the lowest adsorption capacity. N-RHB showed increased adsorption following nitrogen modification, consistent with its increased surface area (320 m^2^ g^−1^), pore volume (0.165 cm^3^ g^−1^), and nitrogen-containing surface functionalities. MN-RHB exhibited a slightly lower adsorption capacity than N-RHB despite its higher BET surface area (480 m^2^ g^−1^); deposition of Fe_3_O_4_ nanoparticles on the carbon surface may have reduced the accessibility of some pore openings.

MN,S-RHB exhibited the highest adsorption capacity among the prepared materials. Its higher uptake was associated with the largest BET surface area (650 m^2^ g^−1^), pore volume (0.325 cm^3^ g^−1^), and the presence of oxygen-, nitrogen-, sulfur-, and iron-containing surface components identified by the characterisation analyses. The experimentally obtained equilibrium capacities reported in the manuscript were 34.2 mg g^−1^ for RHB, 62.4 mg g^−1^ for N-RHB, 54.8 mg g^−1^ for MN-RHB, and 118.6 mg g^−1^ for MN,S-RHB. These results indicate that both the textural properties and surface chemistry of the progressively modified biochars contributed to the observed differences in FLX-Na adsorption.

#### 2.2.4. Adsorption Isotherm Modelling

**Figure 8:**
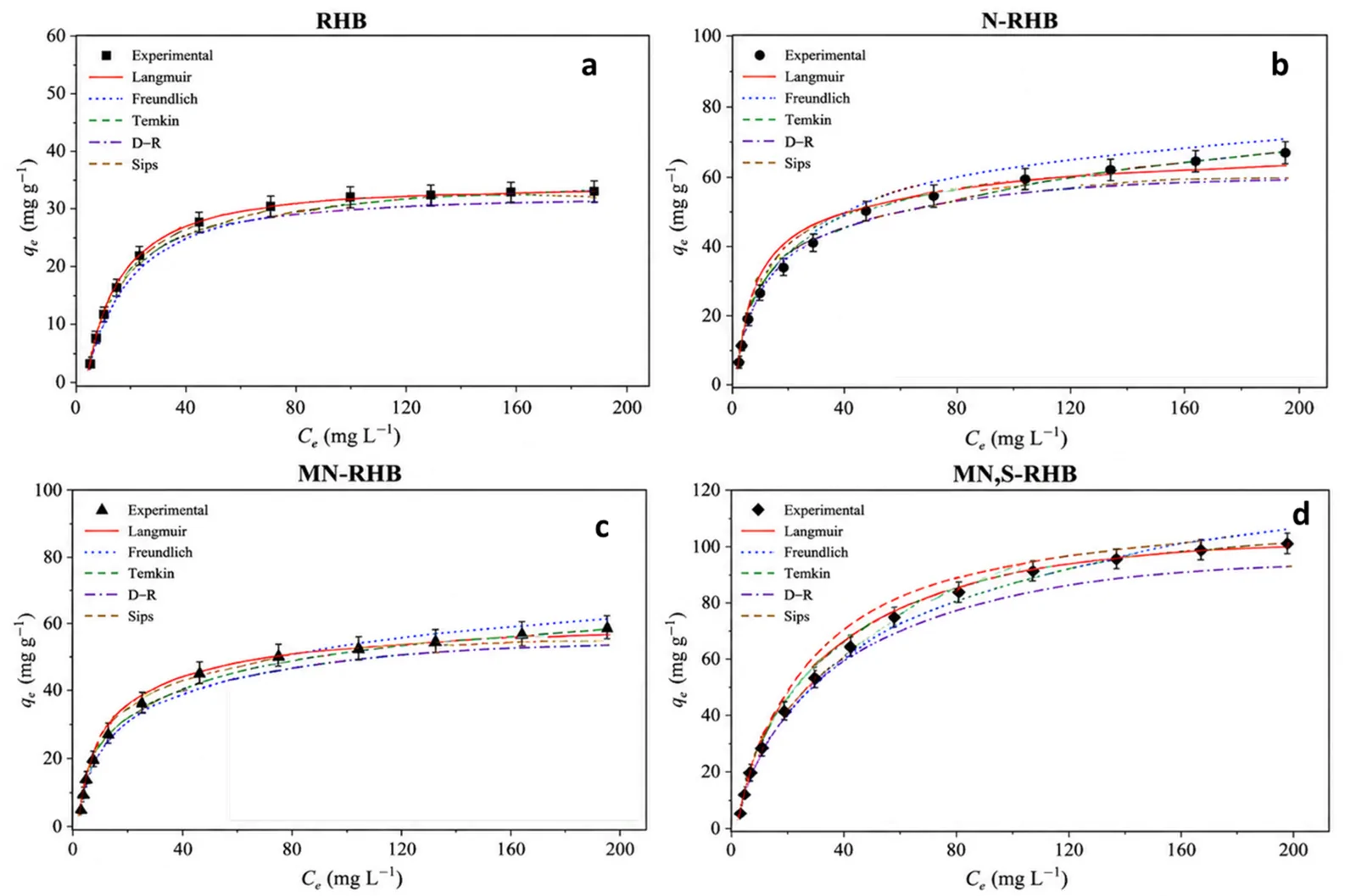
Experimental equilibrium adsorption data and corresponding Langmuir, Freundlich, Temkin, Dubinin-Radushkevich (D-R), and Sips isotherm model fits for FLX-Na adsorption onto (**a**) RHB, (**b**) N-RHB, (**c**) MN-RHB, and (**d**) MN,S-RHB. Numerical model parameters are reported exclusively in Table 4.

The equilibrium adsorption behaviour of FLX-Na on RHB, N-RHB, MN-RHB, and MN,S-RHB was evaluated using the Langmuir, Freundlich, Temkin, Dubinin–Radushkevich (D-R), and Sips isotherm models. The fitted model parameters are reported exclusively in Table 4. Figure 8 is used to present the experimental equilibrium data and the corresponding model-fit curves. The numerical parameters are therefore not repeated in the figure or discussed independently of Table 4.

For all adsorbents, the adsorption capacity increased with increasing initial FLX-Na concentration and gradually approached a plateau at higher concentrations. This behaviour indicates progressive occupation of the available adsorption sites as the FLX-Na concentration increased. The equilibrium adsorption capacities were 34.2, 62.4, 54.8, and 118.6 mg g^−1^ for RHB, N-RHB, MN-RHB, and MN,S-RHB, respectively. Thus, modification of RHB resulted in different equilibrium uptake capacities, with MN,S-RHB showing the highest value under the investigated conditions.

The Langmuir model described the equilibrium data with R^2^ values of 0.982–0.992 for the four adsorbents. The fitted Langmuir maximum capacities and affinity constants were consistent with the experimentally observed adsorption trend. The Langmuir K_L_ values increased from 0.018 L mg^−1^ for RHB to 0.042 L mg^−1^ for MN,S-RHB, indicating differences in the fitted adsorbate-adsorbent affinity among the materials. The Langmuir model provides a useful description of the equilibrium data within the investigated concentration range; however, the fit alone does not establish that adsorption occurs exclusively as an ideal monolayer on a homogeneous surface. The Freundlich model also described the equilibrium data, although its R^2^ values were generally lower than those obtained with the Langmuir and Sips models. The Freundlich parameters indicate differences in the adsorption behaviour of the modified materials and are consistent with changes in surface heterogeneity following nitrogen incorporation and magnetic modification. The Freundlich equation is particularly useful for describing adsorption on energetically non-uniform surfaces, but the fitted parameters should be interpreted as empirical descriptors of the equilibrium data. The Sips model produced R^2^ values of 0.972–0.997, with the highest value obtained for MN,S-RHB. The improved fit obtained for some adsorbents compared with the Langmuir equation indicates that incorporation of the Sips heterogeneity parameter provided a better mathematical description of the equilibrium data. This result is compatible with the heterogeneous surface characteristics indicated by the SEM, FTIR and EDS analyses. The Sips model therefore provides an additional description of the equilibrium behaviour without requiring the surface to be considered ideally homogeneous.

The equilibrium adsorption trend was also considered in relation to the textural properties of the adsorbents. The BET surface area increased from 140 m^2^ g^−1^ for RHB to 320, 480, and 650 m^2^ g^−1^ for N-RHB, MN-RHB, and MN,S-RHB, respectively. The corresponding pore volumes increased from 0.082 cm^3^ g^−1^ for RHB to 0.165, 0.248, and 0.325 cm^3^ g^−1^, respectively. The higher equilibrium uptake of MN,S-RHB therefore occurred together with the largest measured surface area and pore volume. These textural changes can increase the accessible surface available for FLX-Na uptake.

The surface characterisation results provide additional information for interpreting the equilibrium behaviour. SEM showed changes in surface morphology following nitrogen modification and magnetic functionalisation. FTIR and EDS indicated changes in surface chemical composition, while the XRD results confirmed the structural characteristics of the carbon materials and the presence of the Fe_3_O_4_ phase in the magnetic adsorbents. These changes indicate that modification affected both the physical structure and surface composition of the adsorbents. However, the isotherm data alone do not allow the contribution of individual functional groups or specific interactions to be quantitatively separated. An important difference was observed between N-RHB and MN-RHB. Although the BET surface area of MN-RHB (480 m^2^ g^−1^) was higher than that of N-RHB (320 m^2^ g^−1^), its equilibrium adsorption capacity (54.8 mg g^−1^) was lower than that of N-RHB (62.4 mg g^−1^). This difference indicates that adsorption capacity was not determined solely by BET surface area. The deposition of Fe_3_O_4_ on the carbon surface may have altered the accessibility of some pores or adsorption sites.

MN,S-RHB showed the better equilibrium adsorption capacity (118.6 mg g^−1^) together with the highest BET surface area and pore volume. Its surface composition was also altered by the incorporation of nitrogen, sulfur, and Fe_3_O_4_, as indicated by the corresponding characterisation results. The combined changes in pore structure and surface composition provide a basis for the observed difference in equilibrium uptake relative to the other adsorbents. Nevertheless, the relative contribution of pore filling and individual surface interactions cannot be determined solely from the isotherm analysis [31,32,33,34].

### 2.3. Thermodynamic Studies

The influence of temperature on FLX-Na adsorption onto RHB, N-RHB, MN-RHB, and MN,S-RHB was evaluated at 298, 308, and 318 K. For all four adsorbents, the adsorption capacity decreased as the temperature increased, indicating that the adsorption equilibrium shifted towards lower FLX-Na uptake at elevated temperature. This behaviour was more apparent for the modified adsorbents, which showed better adsorption capacities at 298 K. The calculated thermodynamic parameters are presented in Table 5. The values of ΔH° were negative for all adsorbents, ranging from −12.80 to −23.80 kJ mol^−1^, indicating that the adsorption process was exothermic within the investigated temperature range. The magnitude of ΔH° followed the order RHB < MN-RHB < N-RHB < MN,S-RHB. This trend corresponds with the differences observed in the adsorption capacities, although ΔH° should be interpreted as a thermodynamic parameter rather than a direct measure of adsorption capacity.

The calculated ΔG° values were negative at all investigated temperatures, indicating favourable adsorption equilibrium under the corresponding experimental conditions. For RHB, ΔG° changed from −4.72 kJ mol^−1^ at 298 K to −4.18 kJ mol^−1^ at 318 K. The corresponding changes were from −6.53 to −5.71 kJ mol^−1^ for N-RHB, −5.84 to −5.13 kJ mol^−1^ for MN-RHB, and −8.46 to −7.43 kJ mol^−1^ for MN,S-RHB. Thus, ΔG° became less negative with increasing temperature, which is consistent with the experimentally observed reduction in adsorption capacity. The ΔS° values were −27.11, −40.84, −35.44, and −51.48 J mol^−1^ K^−1^ for RHB, N-RHB, MN-RHB, and MN,S-RHB, respectively. The negative values indicate a decrease in the entropy of the adsorption system during establishment of equilibrium. This change may be associated with the transfer and organisation of FLX-Na molecules at the adsorbent-solution interface. However, the entropy data alone do not identify a specific molecular interaction. MN,S-RHB showed the most negative ΔG° values and the largest magnitude of ΔH° among the investigated materials. This behaviour occurred together with its highest equilibrium adsorption capacity and the most developed textural properties, including a BET surface area of 650 m^2^ g^−1^ and pore volume of 0.325 cm^3^ g^−1^. In comparison, RHB exhibited a BET surface area of 140 m^2^ g^−1^ and pore volume of 0.082 cm^3^ g^−1^ [34]. The van’t Hoff plots of ln_Ka_ versus 1/T showed a linear relationship for all four adsorbents (graph is presented in the Supplementary Information as Figure S4). The positive slopes corresponded to negative ΔH° values, supporting the exothermic nature of FLX-Na adsorption. The regression intercepts were used to obtain ΔS°, while the resulting thermodynamic parameters were consistent with the observed decrease in adsorption capacity with increasing temperature.

### 2.4. Xps Analysis Before and After Adsorption

**Figure 9:**
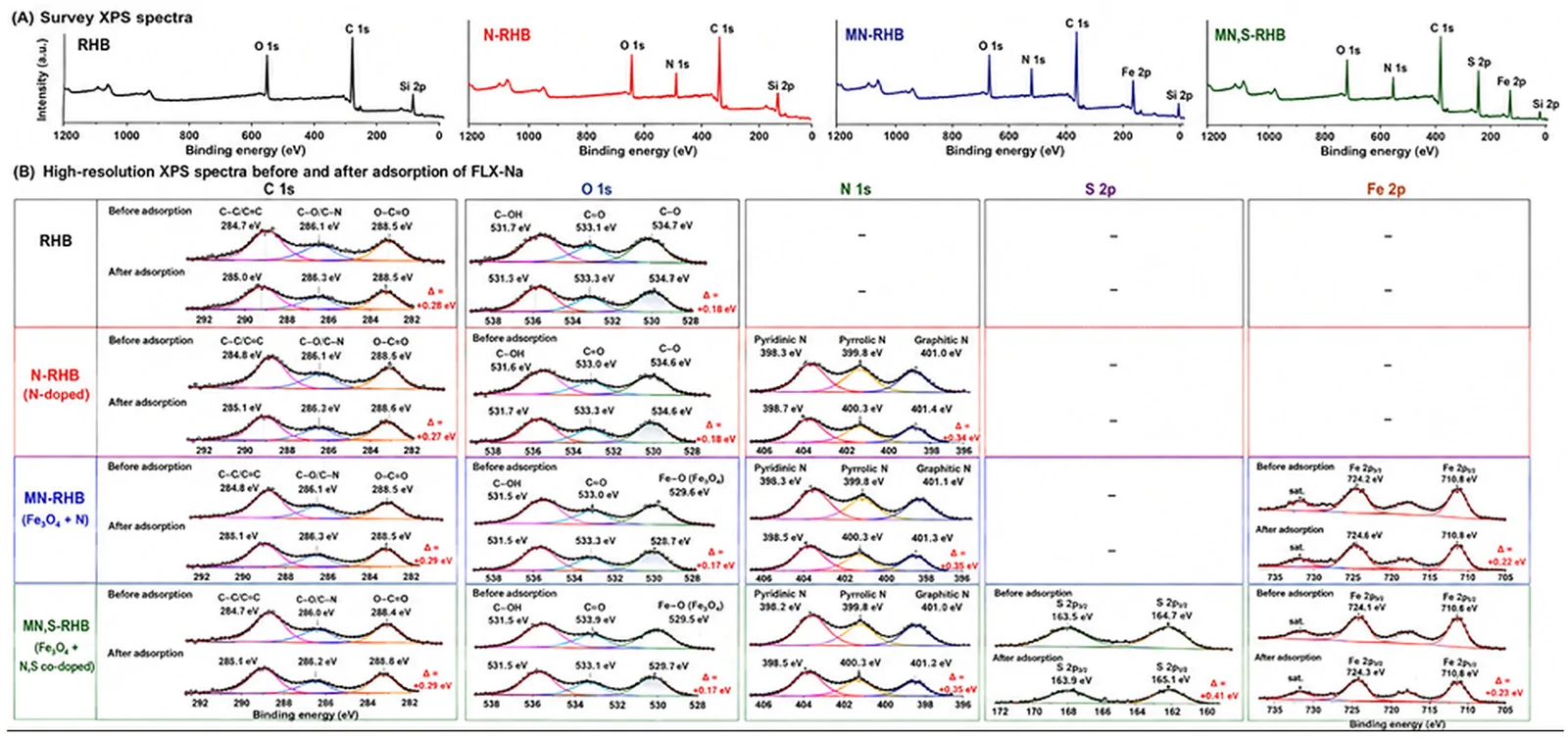
XPS analysis of the adsorbent materials before and after adsorption of FLX-Na.

To investigate the interaction between FLX-Na and the prepared adsorbents, XPS analyses were performed before and after adsorption (Figure 9). Changes in vibrational bands and binding energies were used to evaluate variations in the chemical environment of the surface functional groups following adsorption.

XPS analyses were performed before and after FLX-Na adsorption to evaluate changes in the surface chemical environment of the four adsorbents. The XPS results showed changes in the surface elemental and chemical environments after adsorption. RHB contained mainly C and O, whereas N-RHB additionally showed N. Fe 2p signals were observed for MN-RHB and MN,S-RHB, consistent with the incorporation of the Fe-containing phase during modification. After FLX-Na adsorption, small changes in the binding energies and relative contributions of the C 1s, O 1s, and N 1s components were observed. Changes in the Fe 2p region were also observed for the magnetic adsorbents, while the S 2p region of MN,S-RHB showed changes after adsorption. These observations are consistent with changes at the adsorbent surface following FLX-Na uptake.

The spectroscopic changes are consistent with the batch adsorption results and the modified surface characteristics. The increased surface area and pore volume of the modified adsorbents coincide with higher adsorption capacities, while changes in surface functional-group environments indicate that the surface chemistry changed during FLX-Na uptake. XPS therefore support the involvement of the adsorbent surface in the uptake process. Hydrogen bonding, electrostatic interactions, π-π interactions, surface complexation, and interactions involving Fe-containing sites are plausible contributions based on the observed surface chemistry, but the individual contributions of these interactions cannot be established from FTIR and XPS alone.

### 2.5. Desorption and Regeneration Study

**Figure 10:**
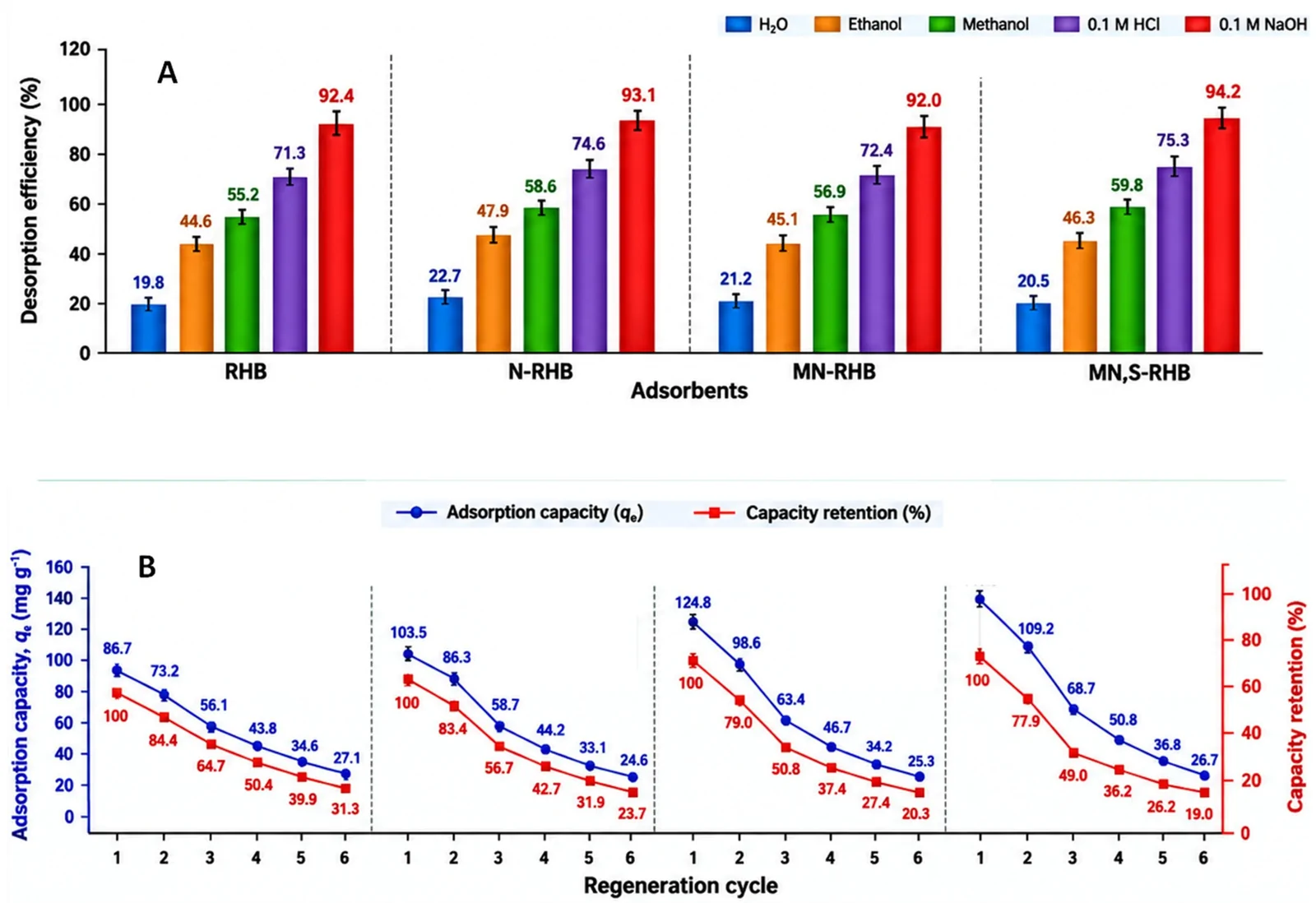
(**A**) Desorption efficiency of FLX-Na from RHB, N-RHB, MN-RHB and MN,S-RHB using different eluents under identical experimental conditions (adsorbent dosage = 0.5 g·L^−1^, initial FLX-Na concentration = 50 mg·L^−1^, solution volume = 50 mL, pH = 6.0, agitation speed = 200 rpm, temperature = 25 ± 1 °C and contact time = 60 min). (**B**) Regeneration performance of the prepared adsorbents using 0.1 M NaOH as the desorbing agent for 06 consecutive adsorption–desorption cycles. Error bars represent the standard deviation of 03 independent experiments.

The regeneration behaviour of the prepared adsorbents was investigated to evaluate their potential for repeated use in FLX-Na removal. Before the regeneration study, different desorbing agents, including deionised water, ethanol, methanol, 0.1 M HCl and 0.1 M NaOH, were evaluated under identical conditions (adsorbent dosage = 0.5 g.L^−1^, initial adsorbed FLX-Na concentration = 50 mg.L^−1^, contact time = 60 min, desorbing solution volume = 50 mL, and temperature = 25 ± 1 °C). The corresponding desorption efficiencies are presented in Figure 10A. Among the investigated eluents, deionised water exhibited the lowest desorption efficiency, ranging from 19.8% to 22.7%, indicating that water alone was insufficient to release the adsorbed FLX-Na molecules. Ethanol and methanol showed moderate desorption efficiencies of 44.6–47.9% and 55.2–59.8%, respectively, whereas 0.1 M HCl improved the desorption efficiency to 71.3–75.3%. The highest desorption efficiencies were obtained using 0.1 M NaOH, reaching 92.4%, 93.1%, 92.0%, and 94.2% for RHB, N-RHB, MN-RHB and MN,S-RHB, respectively. Under alkaline conditions, deprotonation of both the adsorbent surface and FLX-Na molecules weakens hydrogen bonding, electrostatic attraction and surface complexation, thereby facilitating desorption. Similar observations have been reported for regeneration of biochar-based adsorbents used for antibiotic removal, where alkaline eluents generally provide higher desorption efficiencies than neutral or acidic solutions. Therefore, 0.1 M NaOH was selected as the regenerating agent for the subsequent adsorption–desorption cycles.

The regeneration performance of the prepared adsorbents using 0.1 M NaOH is presented in Figure 10B. The regeneration experiments were carried out under adsorption conditions of 50 mg L^−1^ FLX-Na, 0.5 g L^−1^ adsorbent dosage, pH 6.0, contact time of 60 min, and 25 ± 1 °C, followed by desorption using 50 mL of 0.1 M NaOH for 60 min, washing with deionised water until neutral pH, and drying at 60 °C for 12 h before reuse. The adsorption capacities reported in this section therefore correspond to the regeneration experiments and are not directly comparable with the equilibrium capacity obtained from the higher-concentration isotherm experiments. After six adsorption–desorption cycles, MN,S-RHB showed a capacity of 26.7 mg g^−1^, corresponding to 19.0% retention of the reported first-cycle value. The substantial decrease indicates limited reusability under the tested conditions. Similar reductions in adsorption capacity can occur when desorption is incomplete and adsorption sites remain occupied after repeated cycles. Magnetic separation of MN-RHB and MN,S-RHB was also observed during laboratory handling; quantitative magnetic properties are now reported separately from the regeneration experiment.

### 2.6. Effect of Water-Matrix Constituents on Flx-Na Adsorption by Mn,s-Rhb

To further evaluate the practical applicability of MN,S-RHB for FLX-Na removal, the influence of representative aqueous-matrix constituents on the adsorption process was investigated. Although experiments conducted in deionised water provide important information regarding the intrinsic adsorption behaviour of an adsorbent, the presence of inorganic ions and dissolved organic matter in natural and wastewater systems may alter adsorbate-adsorbent interactions and influence the accessibility of adsorption sites. Therefore, additional batch experiments were conducted using controlled aqueous matrices containing representative inorganic ions and dissolved organic matter. MN,S-RHB was selected for the matrix-effect experiments because it exhibited the highest FLX-Na adsorption performance among the adsorbents investigated. These experiments were conducted independently of the equilibrium isotherm experiments under fixed conditions of 50 mg.L^−1^ initial FLX-Na concentration, 0.5 g.L^−1^ adsorbent dosage, 50 mL solution volume, pH 6.0 ± 0.1, 60 min contact time, 250 rpm of stirring, and 25 ± 1 °C. All experiments were performed in triplicate. Deionised water was used as the reference matrix, while additional matrices were prepared by progressively introducing individual inorganic salts, a mixed inorganic electrolyte system, and humic acid.

In this condition, 06 aqueous matrices were investigated which are deionised water (M1), 10 mM NaCl (M2), 5 mM CaCl_2_ (M3), 5 mM MgCl_2_ (M4), a mixed inorganic matrix containing Na^+^, Ca^2+^, Mg^2+^, Cl^−^, SO_4_^2−^ and HCO_3_^−^ (M5), and the same mixed inorganic matrix supplemented with 10 mg.L^−1^ humic acid (M6). The mixed inorganic matrix contained 14 mM Na^+^, 2 mM Ca^2+^, 2 mM Mg^2+^, 14 mM Cl^−^, 2 mM SO_4_^2−^ and 4 mM HCO_3_^−^. This experimental design enabled the effects of individual ions, combined inorganic constituents, and dissolved organic matter to be evaluated under controlled conditions.

The: equilibrium adsorption capacity was calculated using the formula mentioned in the methodology section, Equation (1).

(1) $$q_{e}=\frac{C_{0}-C_{e}}{W}\times V$$

where q_e_ is the equilibrium adsorption capacity (mg.g^−1^), C_0_ and C_e_ are the initial and residual FLX-Na concentrations (mg.L^−1^), respectively, V is the solution volume (L), and m is the mass of MN,S-RHB (g).

#### 2.6.1. Effect of Individual Inorganic Ions

The results of the water-matrix experiments are presented in Table 6. In the fixed conditions employed for the matrix study, MN,S-RHB exhibited an adsorption capacity of 84.60 ± 1.30 mg.g^−1^ in deionised water, corresponding to an FLX-Na removal efficiency of 84.60%. This condition was used as the reference for assessing the influence of the different matrix constituents. The addition of 10 mM NaCl produced only a modest decrease in FLX-Na adsorption. The residual FLX-Na concentration increased from 7.70 mg.L^−1^ in deionised water to 9.35 mg.L^−1^ in the NaCl-containing matrix, while the adsorption capacity decreased to 81.30 ± 1.40 mg.g^−1^. The corresponding removal efficiency was 81.30%, indicating retention of 96.1% of the adsorption capacity obtained in deionised water. Thus, the presence of NaCl produced a matrix effect of only 3.9%.

A larger influence can be observed in the presence of divalent cations. In the 5 mM CaCl_2_ matrix, the residual FLX-Na concentration increased to 11.15 mg.L^−1^ and the adsorption capacity decreased to 77.70 ± 1.50 mg.g^−1^, corresponding to a removal efficiency of 77.70%. The capacity retention was 91.8%, representing an 8.2% matrix effect relative to deionised water. Likewise, the addition of 5 mM MgCl_2_ resulted in a residual FLX-Na concentration of 12.05 mg.L^−1^ and an adsorption capacity of 75.90 ± 1.40 mg.g^−1^. The corresponding removal efficiency was 75.90%, with 89.7% capacity retention and a matrix effect of 10.3%. The reduction observed in the presence of Ca^2+^ and Mg^2+^ compared with Na^+^ may be associated with the stronger influence of divalent ions on the interfacial ionic environment and surface-charge characteristics of MN,S-RHB. These ions can interact with charged surface functionalities and modify the electrostatic environment surrounding the adsorbent. Such changes may subsequently affect the accessibility and affinity of surface sites toward FLX-Na. Nevertheless, the observed decrease cannot be attributed to a single mechanism because ionic strength, ion–surface interactions, and changes in adsorbate–surface interactions may operate simultaneously.

#### 2.6.2. Effect of the Mixed Inorganic Matrix

The combined influence of inorganic constituents was subsequently evaluated using a mixed matrix containing 14 mM Na^+^, 2 mM Ca^2+^, 2 mM Mg^2+^, 14 mM Cl^−^, 2 mM SO_4_^2−^ and 4 mM HCO_3_^−^. In this matrix, the residual FLX-Na concentration increased to 15.90 mg.L^−1^, while the adsorption capacity decreased to 68.20 ± 1.60 mg.g^−1^. The corresponding FLX-Na removal efficiency was 68.20%. Compared with the deionised-water reference, MN,S-RHB retained 80.6% of its adsorption capacity, corresponding to a 19.4% reduction. The reduction compared with the individual electrolyte systems indicates that the simultaneous presence of several inorganic constituents exerted a stronger influence on FLX-Na adsorption than the individual salts investigated separately. The decrease in adsorption capacity may be associated with changes in the ionic environment surrounding MN,S-RHB and modification of the interactions between FLX-Na and the surface functionalities introduced during the N,S functionalization and magnetic modification of the rice-husk-derived carbon. The coexistence of monovalent and divalent ions may also influence the electrostatic environment and accessibility of adsorption sites. Despite this reduction, the adsorption capacity remained 68.20 ± 1.60 mg.g^−1^, demonstrating that MN,S-RHB retained substantial FLX-Na adsorption activity in the presence of multiple inorganic constituents.

#### 2.6.3. Effect of Dissolved Organic Matter

The influence of dissolved organic matter was examined by supplementing the mixed inorganic matrix with 10 mg.L^−1^ humic acid. Under this condition, the residual FLX-Na concentration increased to 19.85 mg.L^−1^, whereas the adsorption capacity decreased to 60.30 ± 1.80 mg g^−1^. The corresponding FLX-Na removal efficiency was 60.30%.

Relative to deionised water, 71.3% of the adsorption capacity was retained in the mixed inorganic/humic-acid matrix, corresponding to an overall matrix effect of 28.7%. This represented the largest reduction in adsorption performance among the investigated aqueous conditions. The higher reduction observed in the presence of humic acid can be attributed to the additional complexity introduced by dissolved organic matter. Humic substances may interact with the MN,S-RHB surface, occupy or partially block accessible adsorption sites, and modify the physicochemical environment of the adsorbent. They may also interact with FLX-Na in the aqueous phase and consequently influence its accessibility to the adsorption sites. Therefore, the decrease observed in the presence of humic acid is likely to result from the combined influence of inorganic constituents and dissolved organic matter rather than from a single adsorption mechanism. Despite the decrease in adsorption performance, MN,S-RHB retained an adsorption capacity of 60.30 ± 1.80 mg.g^−1^ and removed 60.30% of the initial FLX-Na concentration. The retention of substantial adsorption activity under the most complex investigated matrix demonstrates the robustness of MN,S-RHB toward FLX-Na removal in the presence of representative inorganic and organic constituents.

#### 2.6.4. Quantitative Assessment of the Matrix Effect

To compare the adsorption performance under different aqueous conditions, the capacity retention relative to the deionised-water reference was calculated using Equation (2):

(2) $$Capacity\;retention,\;\%=\;\frac{qe,matrix}{qe,\;DI}\;\times 100$$

where q_e_,matrix is the adsorption capacity obtained in the respective matrix and q_e_,DI is the adsorption capacity obtained in deionised water under identical matrix-study conditions.

The matrix effect was calculated according to Equation (3):

(3) $$Matrixeffect,\%=1-\;\frac{qe,matrix}{qe,DI}\;\times 100$$

The calculated matrix effect increased progressively from 3.9% in the NaCl matrix to 8.2% in the CaCl_2_ matrix, 10.3% in the MgCl_2_ matrix, 19.4% in the mixed inorganic matrix, and 28.7% in the mixed inorganic/humic-acid matrix. Correspondingly, the adsorption capacity decreased from 84.60 ± 1.30 mg.g^−1^ in deionised water to 81.30 ± 1.40, 77.70 ± 1.50, 75.90 ± 1.40, 68.20 ± 1.60 and 60.30 ± 1.80 mg.g^−1^, respectively.

The progressive reduction in adsorption capacity with increasing matrix complexity indicates that coexisting aqueous constituents influenced FLX-Na adsorption onto MN,S-RHB under the tested conditions. The matrix effect increased from 3.9% in the NaCl matrix to 28.7% in the mixed inorganic/humic-acid matrix. These results show that ionic composition and dissolved organic matter can alter adsorption performance, while the controlled matrix experiments do not by themselves establish a specific molecular mechanism. MN,S-RHB retained 71.3% of its deionised-water reference capacity in the most complex matrix tested. This observation supports further evaluation in authentic wastewater but should not be interpreted as evidence of field-scale treatment performance.

## 3. Discussion

The progressive changes in surface area, pore volume, surface charge, elemental composition, carbon structure, and Fe loading show that the four materials differ substantially in physicochemical properties. MN,S-RHB combined the largest measured surface area and pore volume with nitrogen-, sulfur-, and iron-containing surface features and the highest equilibrium FLX-Na uptake under the investigated conditions. However, the preparation route for MN,S-RHB also included KOH/H_3_PO_4_ activation and a higher carbonisation temperature than the other materials. The present comparative design therefore does not permit the contribution of N/S co-doping to be isolated from activation, temperature, and magnetic modification effects.

The VSM results provide quantitative evidence of magnetic response for MN-RHB and MN,S-RHB, while ICP-OES confirms substantially greater Fe loading in the magnetic materials than in RHB and N-RHB. Raman and CHNS measurements provide complementary information on the carbon structural environment and bulk elemental composition. The pH_PZC_ and zeta-potential measurements show progressive changes in surface-charge behaviour across the material series. These characterisation results support differences in material properties but do not, individually or collectively, establish a unique molecular adsorption pathway.

The XPS observations indicate changes in the surface chemical environment after FLX-Na uptake. Hydrogen bonding, electrostatic interactions, π-π interactions, surface complexation, and interactions involving Fe-containing sites remain plausible contributions, but their individual contributions cannot be distinguished from the present measurements. Similarly, the higher PSO and Sips coefficients of determination describe empirical model fit and should not be interpreted as proof of chemisorption or a particular surface mechanism.

The regeneration and water-matrix experiments identify practical limitations of the present batch study. The six-cycle retention of 19.0% for MN,S-RHB indicates limited reusability under the tested conditions, and the reported first-cycle regeneration capacity requires verification because it exceeds the mass-balance limit implied by the stated regeneration conditions. The matrix experiments further show that increasing aqueous complexity reduced adsorption capacity, with 71.3% retention in the mixed inorganic/humic-acid matrix. Because the adsorption experiments were conducted mainly at 200 mg L^−1^ FLX-Na and the analytical method was UV-Vis without HPLC confirmation or independent degradation/stability controls, the results are interpreted as adsorption behaviour under controlled laboratory conditions rather than as validated field-scale treatment performance.

## 4. Materials and Methods

### 4.1. Materials

Chemicals Required

Raw rice husk (RH) was obtained from Aquael Zoo (Wrocław, Poland) in 500 g packages. The RH was washed three times with double-distilled water (5000 mL per batch) under vigorous stirring for 30 min, thoroughly rinsed, oven-dried at 373.15 K for 24 h, ground, and sieved to <500 μm. The processed RH was stored in airtight containers until use.

All reagents were of analytical grade and used without further purification. Potassium hydroxide (KOH; >85%, pellets; Merck KGaA, Darmstadt, Germany) and phosphoric acid (H_3_PO_4_; 85%; Sigma-Aldrich Chemie GmbH, Taufkirchen, Germany) were used as activating agents. Urea (99–100.5%) and thiourea (>99%), both obtained from Sigma-Aldrich Chemie GmbH (Taufkirchen, Germany), served as nitrogen and nitrogen/sulfur precursors, respectively. Ferric chloride hexahydrate (FeCl_3_·6H_2_O; 97%) and ferrous sulfate heptahydrate (FeSO_4_·7H_2_O; 99.99%; Sigma-Aldrich Chemie GmbH, Taufkirchen, Germany) were used for magnetic modification. Hydrochloric acid (HCl; 37%) and sodium hydroxide (NaOH; 98%), supplied by VWR International bv (Leuven, Belgium), were used for pH adjustment and washing during adsorption and regeneration experiments. Flucloxacillin sodium (FLX-Na; ≥95%; CAS No. 1847-24-1), obtained from Sigma-Aldrich Chemie GmbH (Taufkirchen, Germany), was used as the model antibiotic.

A 1000 mg L^−1^ FLX-Na stock solution was prepared in double-distilled water and stored at 277.15 K in the dark; working solutions were freshly prepared by dilution. High-purity Nitrogen gas, N_2_ (99.99%) was used to maintain an inert atmosphere during pyrolysis and thermal activation. Acid-washed glassware was thoroughly rinsed with double-distilled water before use.

For the water-matrix experiments, controlled aqueous matrices were prepared to evaluate the influence of selected inorganic electrolytes and dissolved organic matter on FLX-Na adsorption by MN,S-RHB. Deionised water was used as the reference matrix (M1). Individual electrolyte matrices were prepared using 10 mM NaCl (M2), 5 mM CaCl_2_ (M3), and 5 mM MgCl_2_ (M4). A mixed inorganic matrix (M5) was prepared containing 14 mM Na^+^, 2 mM Ca^2+^, 2 mM Mg^2+^, 14 mM Cl^−^, 2 mM SO_4_^2−^, and 4 mM HCO_3_^−^. The sixth matrix (M6) consisted of the same mixed inorganic matrix supplemented with 10 mg L^−1^ humic acid. The water-matrix experiments were conducted using 50 mg L^−1^ FLX-Na, 0.5 g L^−1^ MN,S-RHB, and a solution volume of 50 mL at pH 6.0 ± 0.1. The suspensions were agitated at 250 rpm for 60 min at 25 ± 1 °C. All matrix experiments were performed in triplicate under identical conditions to allow comparison of the influence of individual electrolytes, combined inorganic constituents, and dissolved organic matter on FLX-Na adsorption.

### 4.2. Methodology

Four rice husk-derived adsorbents, namely raw rice husk biochar (RHB), nitrogen-doped rice husk biochar (N-RHB), magnetic nitrogen-doped rice husk biochar (MN-RHB), and magnetic nitrogen/sulfur co-doped rice husk biochar (MN,S-RHB), were synthesised through sequential carbonisation, heteroatom doping, chemical activation, and magnetic functionalisation. The stepwise modification was carried out to examine the influence of each treatment on the physicochemical properties of the resulting materials. Unless otherwise stated, all thermal treatments were performed in a programmable horizontal tubular furnace under a continuous N_2_ atmosphere (99.99%) at a flow rate of 150 mL min^−1^. The furnace temperature was increased at a heating rate of 10 K min^−1^ to the desired temperature and maintained for the specified residence time. After thermal treatment, the samples were allowed to cool naturally to room temperature under a nitrogen atmosphere before further processing.

#### 4.2.1. Synthesis of Raw Rice Husk Biochar (Rhb)

Pretreated rice husk was dried at 378.15 K for 24 h and ground to obtain a particle size of <500 μm. Approximately 10 g of the dried biomass was placed in an alumina crucible and carbonised at 873.15 K for 2 h under a nitrogen atmosphere.

After cooling, the biochar was washed repeatedly with hot double-distilled water, followed by washing with 0.1 M HCl to remove residual inorganic constituents. The material was subsequently rinsed with double-distilled water until neutral pH was attained, dried at 353.15 K for 12 h, and stored in airtight glass containers. The resulting biochar was denoted as RHB [35].

#### 4.2.2. Synthesis of Nitrogen-Doped Rice Husk Biochar (N-Rhb)

Nitrogen-doped biochar was prepared by urea-assisted impregnation followed by pyrolysis. Briefly, 10 g of pretreated rice husk was dispersed in 100 mL of an aqueous solution containing 20 g of urea (0.33 mol). The suspension was magnetically stirred at room temperature for 12 h, after which it was dried at 353.15 K. The impregnated material was carbonised at 873.15 K for 2 h under the same conditions as RHB. The resulting biochar was washed sequentially with 0.1 M HCl and double-distilled water until neutral pH was reached, dried at 353.15 K for 12 h, and stored in airtight containers. The obtained material was designated as N-RHB [36].

#### 4.2.3. Synthesis of Magnetic Nitrogen-Doped Rice Husk Biochar (Mn-Rhb)

Magnetic modification of N-RHB was carried out using an in situ co-precipitation method. Initially, 5 g of N-RHB was dispersed in 200 mL of double-distilled water by ultrasonication for 30 min. Iron(III) chloride hexahydrate (5.40 g, 0.02 mol) and iron(II) sulfate heptahydrate (2.78 g, 0.01 mol) were subsequently added to the suspension, maintaining a Fe^3+^:Fe^2+^ molar ratio of 2:1. The suspension was heated to 353.15 K under continuous stirring (600 rpm) and a nitrogen atmosphere. A freshly prepared 2.0 M NaOH solution was added dropwise until the pH reached approximately 10, promoting the precipitation of iron oxide particles according to:

Fe^2+^ + 2Fe^3+^ + 8OH^−^ → Fe_3_O_4_ + 4H_2_O

The reaction mixture was maintained at 353.15 K for 2 h. The resulting magnetic composite was separated using a permanent magnet, washed repeatedly with double-distilled water and absolute ethanol until neutral pH, and dried at 353.15 K overnight. A subsequent heat treatment at 673.15 K for 1 h under a nitrogen atmosphere was performed before storage. The final material was designated as MN-RHB [37].

#### 4.2.4. Synthesis of Magnetic Nitrogen and Sulfur Co-Doped Rice Husk Biochar (Mn,s-Rhb)

MN,S-RHB was prepared through a combined heteroatom doping, chemical activation, carbonisation, and magnetic modification procedure. Pretreated rice husk (10 g) was impregnated with an aqueous solution containing urea (20 g, 0.33 mol) and thiourea (15 g, 0.20 mol). Potassium hydroxide (10 g, 0.18 mol) and phosphoric acid (10 mL, 85 wt%, 0.146 mol) were added as activating agents, maintaining a complete biomass-to-reagent ratio of approximately 1:4 (*w*/*w*). The mixture was stirred at room temperature for 12 h and subsequently dried at 353.15 K. The dried material was carbonised at 973.15 K for 2 h under a continuous nitrogen atmosphere. After cooling, the carbonised product was washed with 0.1 M HCl followed by repeated washing with double-distilled water until neutral pH was obtained. The cleaned material was then dried at 353.15 K. Magnetic modification was subsequently performed using the same co-precipitation procedure described for MN-RHB. The activated biochar was dispersed in an aqueous solution containing FeCl_3_·6H_2_O (5.40 g, 0.02 mol) and FeSO_4_·7H_2_O (2.78 g, 0.01 mol), followed by the gradual addition of 2.0 M NaOH until the pH reached approximately 10 at 353.15 K under a nitrogen atmosphere. The magnetic product was separated using a permanent magnet, washed repeatedly with double-distilled water and ethanol until neutral pH, dried at 353.15 K for 12 h, and subjected to a final thermal treatment at 673.15 K for 1 h under nitrogen. The resulting material was designated as MN,S-RHB [38].

**Scheme 1:**
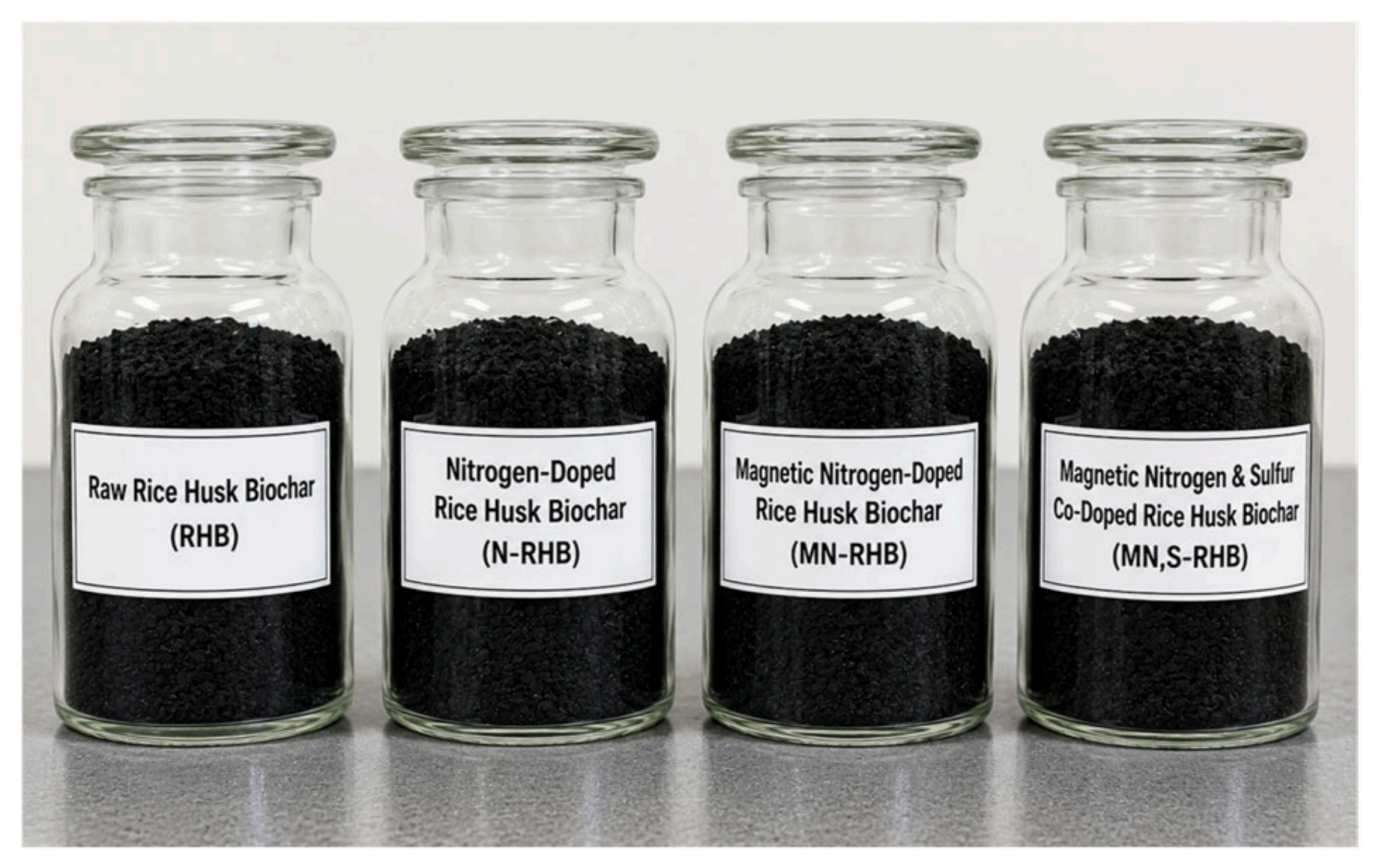
Representative photograph of the prepared rice husk biochar-derived adsorbents following synthesis and drying. From left to right: raw rice husk biochar (RHB), nitrogen-doped rice husk biochar (N-RHB), magnetic nitrogen-doped rice husk biochar (MN-RHB), and magnetic nitrogen and sulfur co-doped rice husk biochar (MN,S-RHB), stored in airtight glass containers before physicochemical characterisation and adsorption experiments.

All adsorbents were synthesised in triplicate under identical experimental conditions. The prepared materials were stored in airtight desiccators at room temperature until further characterisation and adsorption experiments. Images of the developed adsorbents are illustrated in Scheme 1.

### 4.3. Characterisation of Adsorbent Materials

The morphological, structural, textural, and surface-chemical properties of raw rice husk biochar (RHB), N-doped RHB (N-RHB), magnetic N-doped RHB (MN-RHB), and magnetic N/S co-doped RHB (MN,S-RHB) were characterised using complementary techniques. Surface morphology and elemental distribution were examined by FE-SEM/EDS. Crystalline phases were identified by XRD. N_2_ adsorption–desorption isotherms were measured at 77 K after vacuum degassing at 423.15 K for 3 h; specific surface area was calculated by the BET method and pore characteristics by DFT analysis. Surface functional groups were identified by FTIR spectroscopy over 4000–500 cm^−1^. Raman spectroscopy was used to assess the carbon structural environment, with the ID/IG ratio calculated from the D and G bands. Bulk C, H, N and S contents were determined by CHNS elemental analysis. The point of zero charge (pH_PZC_) was determined by the pH-drift method, and zeta-potential measurements were used to estimate the isoelectric point from the pH at which the zeta potential crossed zero. The magnetic properties of MN-RHB and MN,S-RHB were evaluated by vibrating sample magnetometry (VSM), including saturation magnetization (M_s_), remanent magnetization (M_r_), and coercivity (Hc). Total Fe loading was quantified by ICP-OES. XPS measurements were performed using the Thermo Scientific K-Alpha spectrometer described above, with survey and high-resolution C 1s, O 1s, N 1s, S 2p, and Fe 2p spectra acquired for pristine and spent adsorbents. All characterisation measurements were performed using freshly prepared samples, with replicate measurements where applicable.

The point of zero charge (pH_PZC_) was determined by the pH-drift method using 0.04 g adsorbent in 20 mL double-distilled water adjusted to initial pH 2–12 with 0.1 M HNO_3_ and NaOH, followed by equilibration for 24 h at room temperature. The pH_PZC_ was obtained from the intersection of the ΔpH (= pH_final_ − pH_initial_) versus initial-pH curve with ΔpH = 0. Zeta-potential measurements were conducted over the same pH range to determine the surface-charge response; the isoelectric point was estimated from the pH at which the zeta potential crossed zero.

Changes in surface chemistry after FLX-Na adsorption were assessed by FTIR and XPS. XPS measurements were performed using a Thermo Scientific K-Alpha spectrometer equipped with a monochromatic Al Kα source (hν = 1486.6 eV); survey and high-resolution C 1s, O 1s, N 1s, S 2p, and Fe 2p spectra were acquired for pristine and spent adsorbents to evaluate changes in surface composition and chemical states.

All characterisation measurements were performed using freshly prepared samples. Analyses were conducted in triplicate where applicable to assess measurement reproducibility.

For the additional characterisation, Raman spectra were evaluated using the D- and G-band region, CHNS analysis was used to determine bulk elemental composition, VSM hysteresis curves were used to obtain M_s_, M_r_ and Hc for the magnetic adsorbents, zeta-potential measurements were used to determine the isoelectric points, and ICP-OES was used to quantify total Fe loading.

### 4.4. Batch Adsorption Studies

Batch adsorption experiments were conducted to examine the adsorption of Flucloxacillin sodium (FLX-Na) onto raw rice husk biochar (RHB), nitrogen-doped rice husk biochar (N-RHB), magnetic nitrogen-doped rice husk biochar (MN-RHB), and magnetic nitrogen/sulfur co-doped rice husk biochar (MN,S-RHB). The effects of solution pH, contact time, initial FLX-Na concentration, temperature, and adsorbent dosage were assessed. Desorption and regeneration experiments were also performed to assess adsorbent reusability. A 1000 mg L^−1^ FLX-Na stock solution was prepared by dissolving 500 mg FLX-Na in 500 mL of double-distilled water. The pH was adjusted using 0.1 M HCl or 0.1 M NaOH, and working solutions were freshly prepared by appropriate dilution before each experiment.

The amount of FLX-Na adsorbed at equilibrium, q_ea_ (mg.g^−1^), was calculated using the mass balance equation (Equation (4)):

(4) $$q_{ea}=\;\frac{C_{i}-\;C_{e}}{W}\;\times V$$

where C_i_: initial concentration; Ce: final concentration; V: volume in L; and W: weight of the adsorbent in g. The q_ea_—adsorption capacity, mg.g^−1^.

Working solutions were freshly prepared from the 1000 mg L^−1^ FLX-Na stock solution by appropriate dilution. For contact-time experiments, 250 mL of 200 mg L^−1^ FLX-Na solution was mixed with 0.5 g of adsorbent and agitated at 200 rpm in a temperature-controlled water-bath shaker at 298 K; aliquots were collected at predetermined intervals. The effect of pH was evaluated using 20 mL of 200 mg L^−1^ FLX-Na solution, with the initial pH adjusted from 2 to 12 using 0.1 M HNO_3_ or 0.1 M NaOH, followed by addition of 0.04 g adsorbent and agitation at 200 rpm for 2 h at 298 K. After adsorption, samples were filtered through 0.45 μm nylon syringe filters (Fisherbrand, 25 mm), and residual FLX-Na concentrations were determined by UV–Vis spectrophotometry. The λmax of FLX-Na was determined by scanning a 10 mg L^−1^ standard solution from 200–400 nm and was identified at 274 nm. Quantification was performed at 274 nm using a calibration curve prepared over 5–200 mg L^−1^; the calibration graph is presented in the Supplementary Information as Figure S1.

#### 4.4.1. Adsorption Kinetic Equation

To explore the adsorption behaviour of RHB, N-RHB, MN-RHB and MN,S-RHB, pseudo-first-order kinetics and pseudo-second-order kinetics equations were fitted to their adsorption curve. Equation (5) was as follows:

(5) $$\log\left(q_{e}-q_{t}\right)=\log q_{e}-\frac{k_{1}}{2.303}t$$

where k_1_ (per h) is the pseudo-first-order rate constant, q_e_ and q_t_ (mg.g^−1^) are the amount of FLX-Na adsorbed at equilibrium and time, respectively.

Equation (6) used in pseudo-second-order kinetics [38,39,40,41],

(6) $$\frac{t}{q_{t}}=\frac{1}{k_{2}q_{e}^{2}}+\frac{1}{q_{e}}t$$

where q_e_ and q_t_ (mg.g^−1^) are the amounts of adsorption at any time (t, h), k_2_ (g.mg.h^−1^) is the pseudo-second-order adsorption rate constant, and R^2^ -correlation coefficients are determined by plotting t/qt against t.

The equation used for intra-particle diffusion is given in Equation (7):

(7) $$q=k_{id}t^{0.5}+C$$

where kid (mg.g^−1^.min^−1/2^) is the intra-particle diffusion rate constant, and C (mg.g^−1^) is a constant in the intra-particle diffusion model, which implies the consequences of the boundary layer on mass transfer effect.

#### 4.4.2. Adsorption Isotherm and Thermodynamic Studies

At the same time, to investigate and extract the information on adsorption behaviours of antibiotics onto the synthesized adsorbents, two different adsorption models, namely Langmuir [35] and Freundlich, were inspected.

In both models, the experimental data were expressed through the plots of q_e_ Vs C_e_ and the values of both parameters were calculated from the fitting equations described below. The Freundlich isotherm is signified by the following Equation (8):

(8) $$\mathrm{Log}q_{e}=\log K_{f}+\;\left(\frac{1}{n}\right)\;logC_{e}$$

where C_e_ (mg∙L^−1^) is the equilibrium concentration, qe (mg∙g^−1^) is the amount adsorbed at a specific amount of adsorbent at equilibrium, K_f_ (mg∙g^−1^) is the constant which is the adsorption capacity, and n (mg∙g^−1^) is a constant which is the adsorption capacity. Equation (6) is as follows:

Whereas the Langmuir isotherm equation is signified in the following form of Equation (9):

(9) $$\;\frac{{\;C}_{e}}{q_{e}}=\;\frac{1}{q_{max}b}+\;\frac{C_{e}}{q_{max}}$$

where qe (mg g^−1^) is the FLX-Na amount adsorbed at equilibrium, Ce (mg L^−1^) is the equilibrium concentration, q_max_ is the Langmuir constant linked to the adsorption capacity, and b (L.mg^−1^) is also a Langmuir constant related to the energy of adsorption.

Dubinin-Radushkevich (D-R) isotherm

The Dubinin-Radushkevich (D-R) isotherm model was additionally applied to evaluate the adsorption energy characteristics and heterogeneity of the adsorption process. The nonlinear form of the D-R equation is expressed as Equation (10):

(10) qe=qmexp(−βε2)

where (qe) (mg·g^−1^) is the equilibrium adsorption capacity, (q_m_) (mg·g^−1^) is the theoretical saturation capacity, β (mol^2^·kJ^−2^) is the D-R isotherm constant related to adsorption energy, and ε is the Polanyi potential calculated using Equation (11):

(11) $$\varepsilon \;=RTIn\;(1+(\frac{1}{Ce})$$

The mean adsorption energy (E) was determined according to Equation (12):

(12) $$E=\frac{1}{\surd 2\beta }$$

where the magnitude of (E) provides insight into the adsorption mechanism, where values below approximately 8 kJ·mol^−1^ are generally associated with physisorption processes.

Sips Isotherm

The Sips model combines the characteristics of the Langmuir and Freundlich models and is suitable for describing adsorption on heterogeneous surfaces over a wide concentration range. Equation (13) is as follows

(13) qe={qs(Ks∗Ce)^n}/{1+(Ks∗Ce)^n}

where q_s_ (mg.g^−1^) is the maximum adsorption capacity, Ks (L.mg^−1^) is the Sips equilibrium constant, and n is the heterogeneity factor.

The thermodynamic behaviour of FLX-Na adsorption onto the synthesised adsorbents was evaluated using equilibrium adsorption data obtained at 298, 308, and 318 K. The standard thermodynamic parameters, including Gibbs free energy change (ΔG°), enthalpy change (ΔH°), and entropy change (ΔS°), were determined to assess the temperature dependence of the adsorption process. These parameters were calculated using Equations (14)–(16) [41]:

(14) ΔG°=ΔH°−TΔS°

(15) ΔG°=−RTInKa

(16) $$InKa=\frac{\Delta S\circ }{R}-\frac{\Delta H\circ }{RT}$$

where R is a gas constant (8.314 J·mol^–1^·K^–1^), T (K) is the value of temperature, and K_a_ is the thermodynamic equilibrium constant. K_a_ is dimensionless, calculated by converting the Langmuir equilibrium constant (b) in units of L·mol^−1^. Isotherm data were sensibly explained with the Langmuir model; it is also reasonable to use the Langmuir equilibrium constant for the determination of ΔG°. The values of ΔG°, ΔH°, and ΔS° were deduced from the Van’t Hoff plot involving lnKa against 1/T.

### 4.5. Desorption and Reusability

Desorption and regeneration tests were performed to assess adsorbent reusability. NaOH, NaCl, H_3_PO_4_, HNO_3_, HCl, and double-distilled water were screened as desorbing media for FLX-Na. After adsorption, the spent adsorbent was recovered by filtration or magnetic separation, rinsed with double-distilled water, and contacted with the selected desorption medium under the same experimental conditions. The regenerated material was washed to neutral pH, dried at 353.15 K, and reused for subsequent adsorption cycles. Adsorption—desorption cycles were continued until the adsorption capacity decreased below 50% of the initial value, with the capacity retention after each cycle used to assess regeneration performance.

The desorption capacity (q_ed_) was calculated using Equation (17).

(17) $$q_{ed}=C_{ed}\times \frac{V}{w}$$

where C_ed_ is the absorbance of the desorption solution, V is the volume in L, and W is the weight of the exhausted adsorbent in g.

The percentage desorption was calculated by using the given formula in Equation (18).

(18) $$\%\;Desorption=\frac{q_{ed}}{q_{ea}}\times 100$$

where q_ed_ is the desorption capacity, and q_ea_ is the adsorption capacity (mg g^−1^).

### 4.6. Statistical Analysis

All adsorption experiments were performed in triplicate under identical conditions to ensure reproducibility. The reported adsorption capacities and removal efficiencies correspond to the mean ± standard deviation (SD) of three independent measurements. Standard deviations were calculated from experimental replicate values, and error bars in the figures represent ± SD from triplicate experiments (n = 3).

### 4.7. Xps Validation Before and After Adsorption

XPS analyses were performed on pristine and FLX-Na-loaded adsorbents to elucidate changes in surface chemistry associated with adsorption. XPS measurements were conducted using a Thermo Scientific K-Alpha spectrometer (Thermo Fisher Scientific, Waltham, MA, USA) with a monochromatic Al Kα source (hν = 1486.6 eV). Survey and high-resolution C 1s, O 1s, N 1s, S 2p, and Fe 2p spectra were collected and deconvoluted to examine changes in surface composition and chemical states following adsorption. Comparative FTIR/XPS analysis was used to assess the possible involvement of oxygen-, nitrogen-, and sulfur-containing functionalities and Fe_3_O_4_-associated sites in FLX-Na uptake, with particular consideration of electrostatic interactions, hydrogen bonding, π-π interactions, surface complexation, and pore filling.

## 5. Conclusions and Future Perspectives

This study investigated the adsorption of flucloxacillin sodium (FLX-Na) using rice-husk-derived biochars with progressively different surface characteristics. The four preparation routes produced materials with distinct textural and surface properties, and MN,S-RHB showed the highest equilibrium adsorption capacity (118.6 mg g^−1^) under the investigated conditions. Because chemical activation and a higher carbonisation temperature were introduced only for MN,S-RHB, the present design does not allow the contribution of N/S co-doping to be isolated from the effects of activation, temperature, and magnetic modification. The kinetic data were best described by the pseudo-second-order model, while the Sips model gave the highest R^2^ among the tested equilibrium models. Thermodynamic analysis gave negative ΔG° and ΔH° values, indicating favourable, exothermic adsorption within the investigated temperature range. XPS changes were consistent with alterations in the surface chemical environment after FLX-Na uptake, but the individual contributions of specific interactions could not be distinguished from these measurements alone. Regeneration with 0.1 M NaOH resulted in a substantial decrease in adsorption capacity over six cycles. Quantitative VSM analysis showed magnetic hysteresis for MN-RHB and MN,S-RHB, while Raman, CHNS, zeta-potential, and ICP-OES measurements provided additional information on carbon structure, bulk elemental composition, surface charge, and Fe loading, respectively.

The water-matrix experiments showed that adsorption capacity decreased as the aqueous phase became more chemically complex. For MN,S-RHB, the capacity decreased from 84.60 ± 1.30 mg g^−1^ in deionised water to 60.30 ± 1.80 mg g^−1^ in the mixed inorganic/humic-acid matrix, corresponding to 71.3% capacity retention. These results indicate that coexisting inorganic constituents and dissolved organic matter can influence FLX-Na uptake under the tested conditions. The study was conducted mainly at elevated FLX-Na concentrations and with controlled matrices.

Future studies should extend these findings by evaluating the adsorbents in authentic pharmaceutical, hospital, and other wastewater streams containing multiple coexisting contaminants. Continuous-flow column experiments would also be useful for determining breakthrough behaviour and dynamic adsorption performance under conditions more representative of practical treatment systems. Validated chromatographic analysis, appropriate stability controls, nonlinear kinetic and equilibrium regression, and additional material characterisation can further strengthen assessment of the adsorption and reuse behaviour of the modified biochars.

## Figures and Tables

**Table 1:** Features of the selected β-lactam antibiotics.

Compound Name	Chemical Structure	Molecular Formula	Molecular Weight (g.mol^−1^)	Wavelength (nm)
Flucloxacillin sodium (FLX-Na)	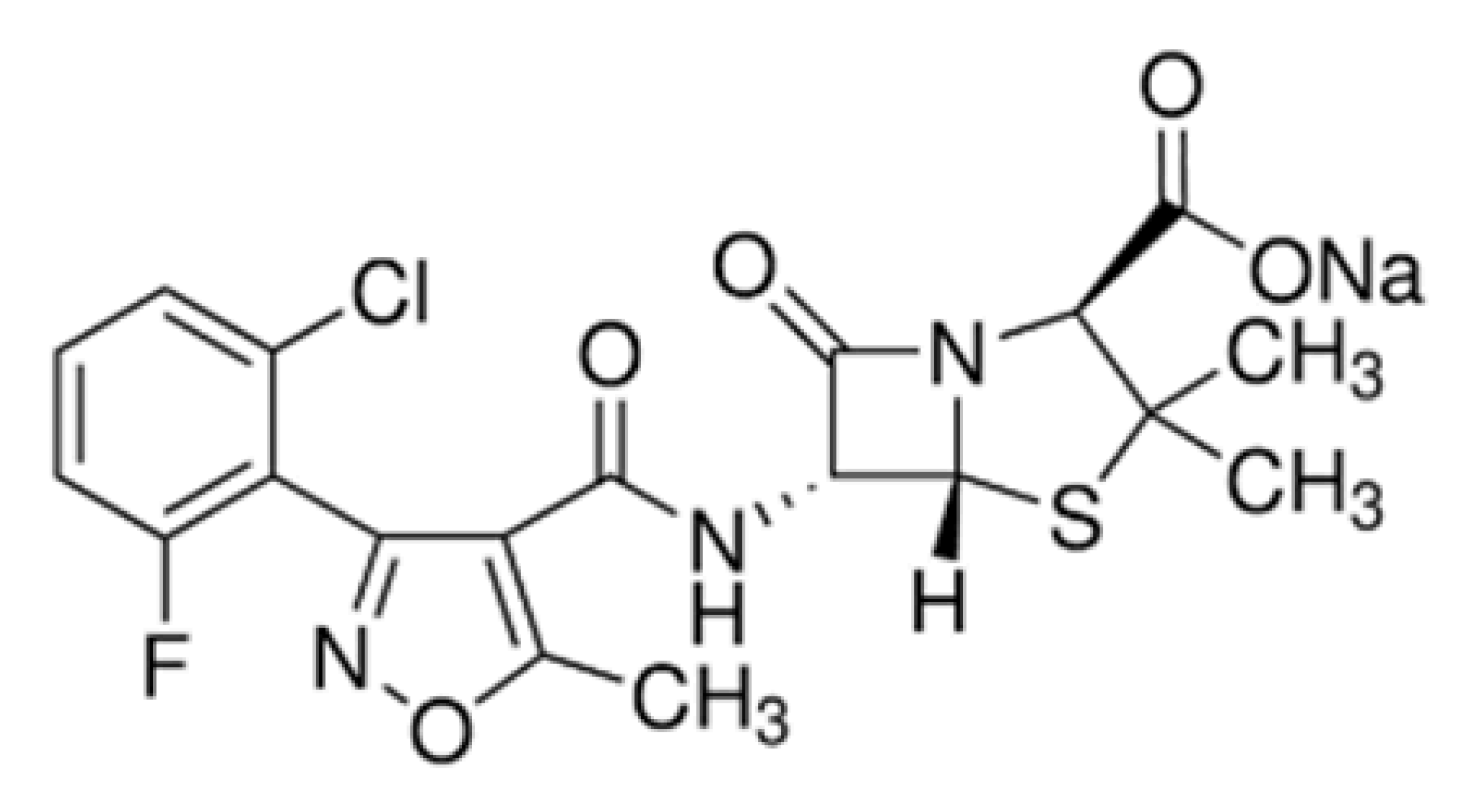	C_19_H_16_ClFN_3_O_5_S.Na	475.85	274

**Table 3:** Kinetic parameters for FLX-Na adsorption onto different adsorbents.

Adsorbent	Experimental q_e_ (mg.g^−1^)	PFO q_e_ (mg.g^−1^)	K_1_ (min^−1^)	R^2^ (PFO)	PSO qe (mg.g^−1^)	K_2_ (g.mg^−1^.min^−1^)	R^2^ (PSO)	α (mg.g^−1^.min^−1^)	β (g m.^−1^)	R^2^ (Elovich)	k_id_ (mg.g^−1^.min^−^½)	C	R^2^ (ID)
RHB	32.8	28.5	0.015	0.921	33.4	0.0028	0.991	4.18	0.153	0.945	2.48	6.4	0.931
N-RHB	59.6	53.1	0.022	0.944	60.3	0.0045	0.995	9.64	0.097	0.963	3.92	11.2	0.946
MN-RHB	52.2	47.6	0.018	0.937	53.1	0.0039	0.993	8.23	0.104	0.958	3.61	9.6	0.939
MN,S-RHB	112.7	103.2	0.031	0.958	114.4	0.0084	0.998	21.85	0.061	0.982	6.88	18.4	0.968

**Table 4:** Equilibrium isotherm parameters for FLX-Na adsorption onto RHB, N-RHB, MN-RHB, and MN,S-RHB obtained using Langmuir, Freundlich, Temkin, Dubinin-Radushkevich (D-R), and Sips models.

Adsorbent	Langmuir q_max_ (mg g^−1^)	K_L_ (L mg^−1^)	R^2^	Freundlich K_F_	1/n	R^2^	Temkin K_T_ (L g^−1^)	b_T_ (J mol^−1^)	R^2^	D-R q_m_ (mg g^−1^)	E (kJ mol^−1^)	R^2^	Sips q_s_ (mg g^−1^)	K_s_ (L mg^−1^)	n	R^2^
**RHB**	34.2	0.018	0.982	9.8	0.72	0.935	1.72	362	0.943	31.7	6.81	0.921	34.8	0.019	0.84	0.972
**N-RHB**	62.4	0.031	0.988	24.5	0.58	0.952	3.41	426	0.961	58.2	9.81	0.948	63.7	0.031	0.89	0.983
**MN-RHB**	54.8	0.026	0.985	19.2	0.61	0.948	2.96	411	0.955	51.5	8.84	0.942	55.9	0.028	0.86	0.978
**MN,S-RHB**	118.6	0.042	0.992	48.6	0.43	0.964	6.88	548	0.983	115.3	11.79	0.975	102	0.046	0.94	0.997

**Table 5:** Thermodynamic parameters and values corresponding to them.

Adsorbent	T (K)	K°	ΔG° (kJ mol^−1^)	ΔH° (kJ mol^−1^)	ΔS° (J mol^−1^ K^−1^)
**RHB**	298	6.720	−4.72	−12.80	−27.11
	308	5.682	−4.45		
	318	4.856	−4.18		
**N-RHB**	298	13.952	−6.53	−18.70	−40.84
	308	10.920	−6.12		
	318	8.679	−5.71		
**MN-RHB**	298	10.561	−5.84	−16.40	−35.44
	308	8.518	−5.49		
	318	6.965	−5.13		
**MN,S-RHB**	298	30.406	−8.46	−23.80	−51.48
	308	22.259	−7.95		
	318	16.617	−7.43		

**Table 6:** Effect of aqueous-matrix composition on FLX-Na adsorption by MN,S-RHB.

Aqueous Matrix	C_e_ (mg.L^−1^)	q_e_ (mg.g^−1^)	Removal Efficiency (%)	Capacity Retention (%)	Matrix Effect (%)
Deionised water	7.70	84.60 ± 1.30	84.60	100.0	0.0
10 mM NaCl	9.35	81.30 ± 1.40	81.30	96.1	3.9
5 mM CaCl_2_	11.15	77.70 ± 1.50	77.70	91.8	8.2
5 mM MgCl_2_	12.05	75.90 ± 1.40	75.90	89.7	10.3
Mixed inorganic matrix	15.90	68.20 ± 1.60	68.20	80.6	19.4
Mixed inorganic + 10 mg.L^−1^ humic acid	19.85	60.30 ± 1.80	60.30	71.3	28.7

## Data Availability

The raw data supporting the conclusions of this article will be made available by the authors on request.

## Supplementary Materials

The following supporting information can be downloaded at: https://www.mdpi.com/article/10.3390/ijms27209082/s1.
