# Supplementary material for: N, S-Functionalized Magnetic Rice Husk Biochar for Flucloxacillin Sodium Adsorption: Regeneration and Water-Matrix Effects
Source: Int J Mol Sci. 2026 Oct 13;27(20):9082. doi: 10.3390/ijms27209082 (PMC13705566; doi:10.3390/ijms27209082)
Supplement: Supplementary file 1 [file ijms-27-09082-s001.zip › ijms-4563568-supplementary.pdf]

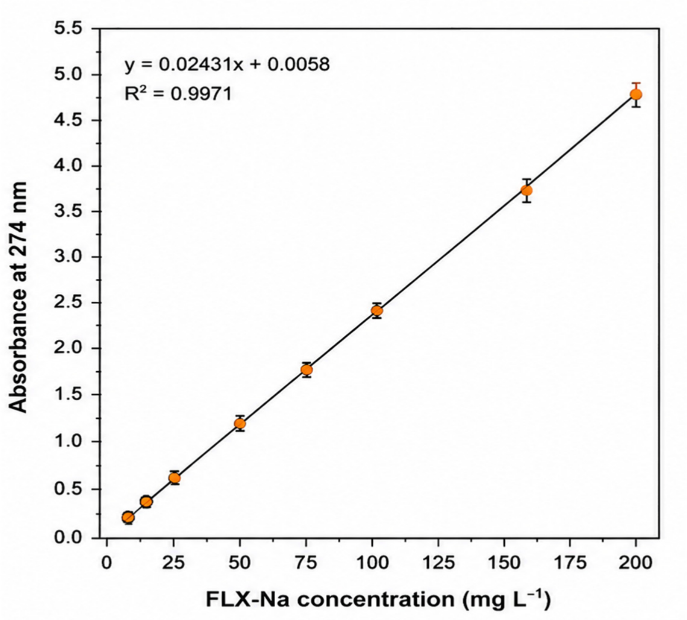

Figure SI-S1: Calibration curve of FLX-Na measured at 274 nm over the concentration range of 5-200 mg L^{-1}. Data points represent the mean absorbance ± standard deviation of three independent measurements (n = 3). The solid line represents the linear regression fit.

SI- Table S1. Textural and structural characteristics of RHB, N-RHB, MN-RHB, and MN,S-RHB obtained from N_2 adsorption–desorption analysis at 77 K and XRD analysis. S_{BET}, total pore volume, Vads and 4V/S represent the measured textural parameters, while $2\theta_{002}$ and d_{002} describe the position and interlayer spacing of the carbon (002) reflection, respectively. The Fe_3O_4 phase was identified from its characteristic diffraction reflections in the magnetically modified adsorbents.

**Sample**	**S_{BET} (m^2 g^{-1})**	**Total pore volume (cm^3 g^{-1})**	**Average pore diameter* (nm)**	**Vads at P/P_0 = 0.99 (cm^3 g^{-1} STP)**	**4V/S (nm)**	**Hysteresis type**	**$2\theta_{002}$ (°)**	**d_{002} (Å)**	**Fe_3O_4 phase**
**RHB**	140	0.082	2.3	53	2.34	H4	27.0	3.30	Absent
**N-RHB**	320	0.165	2.1	107	2.06	H4	27.2	3.27	Absent
**MN-RHB**	480	0.248	2.1	161	2.07	H4	27.3	3.26	Present

**MN,S-RHB**	650	0.325	2.0		210	2.00	H4		27.5	3.24	Present

SI- Table S2. FTIR peak positions, corresponding vibrational assignments, and associated elements of RHB, N-RHB, MN-RHB, and MN,S-RHB.

**Wavenumber (cm^{-1})**	**Assignment**	**Main elements**
**3380**	O-H/N-H stretching	O, H / N, H
**2765**	C-H stretching	C, H
**1615**	N-H/C=N-related vibration	N, H, C
**1550**	C=O/C=C-related vibration	C, O
**1390**	C-H bending/deformation	C, H
**1050**	C-O/C-O-C and C-N vibrations	C, O, N
**610**	Fe-O vibration	Fe, O

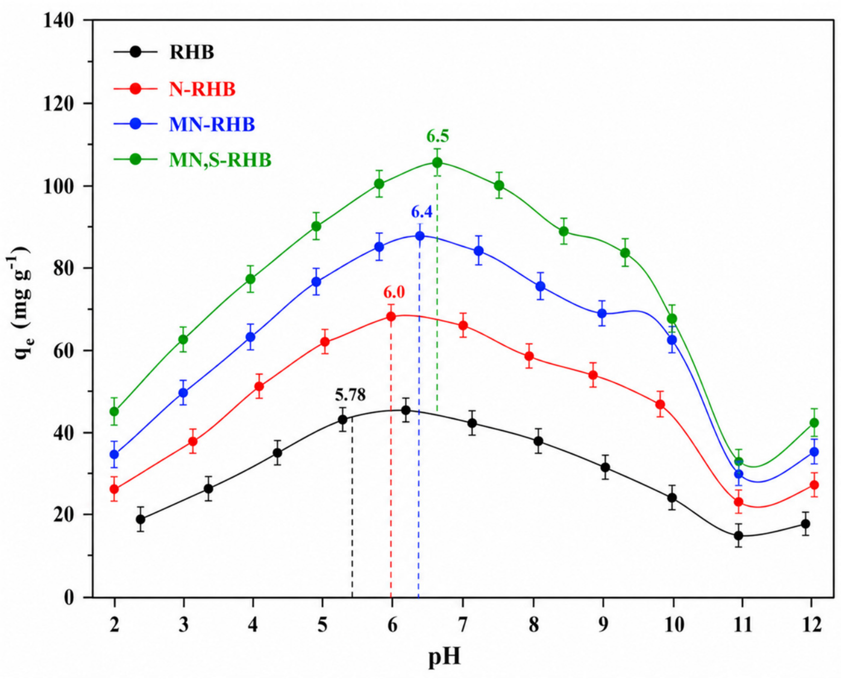

Figure SI-S2: Effect of pH on the adsorption capacity of materials.

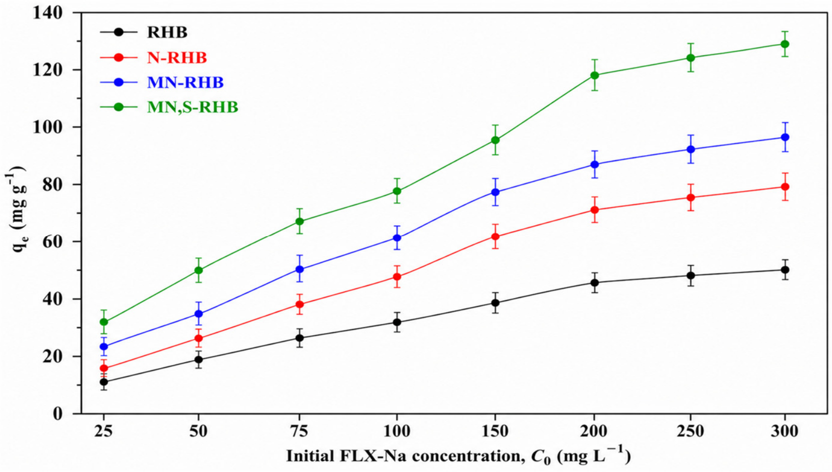

Figure SI-S3: Effect of initial concentration on the adsorption capacity of materials.

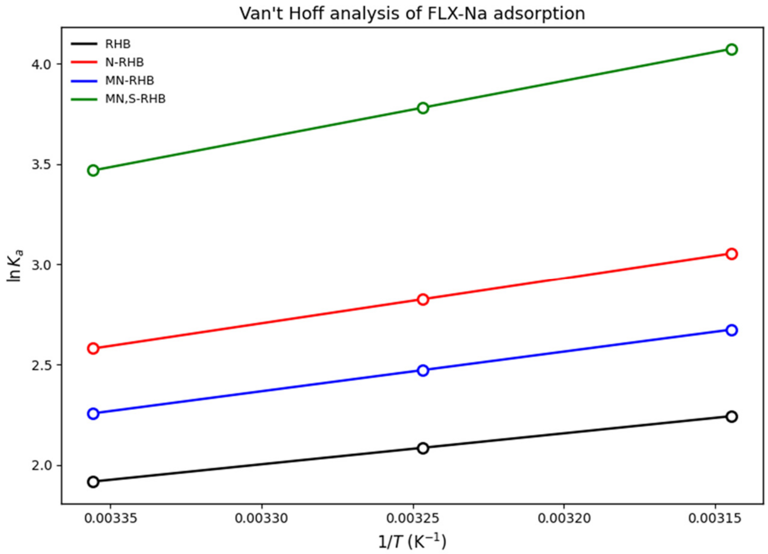

Figure SI-S4: Van't Hoff plots for FLX-Na adsorption onto RHB, N-RHB, MN-RHB, and MN,S-RHB at 298-318 K.
